# Design, synthesis, and anticancer activity of three novel palbociclib derivatives

**DOI:** 10.3389/fonc.2022.959322

**Published:** 2022-08-25

**Authors:** Tian Li, An-Di Zhou, Li-Fei Bai, Xiao-Yang Zhang, Yu-Ting Zhou, Hai-Li Yang, Le-Tian Xu, Xin-Qin Guo, Xi-Yu Zhu, Dong-Jin Wang, Hong-Wei Gu, Xiao-Ming Wang

**Affiliations:** ^1^ Department of Cardio-Thoracic Surgery, State Key Laboratory of Pharmaceutical Biotechnology, Affiliated Drum Tower Hospital, Medical School of Nanjing University, School of Life Sciences, Nanjing University, Nanjing, China; ^2^ Jiangsu Key Laboratory of Biofunction Molecule, School of Life Science and Chemical Engineering, Jiangsu Second Normal University, Nanjing, China; ^3^ Central Laboratory, Nanjing Integrated Traditional Chinese and Western Medicine Hospital Affiliated with Nanjing University of Chinese Medicine, Nanjing, China

**Keywords:** palbociclib derivatives, anticancer activity, CDK4/6, 10-hydroxy camptothecin, Topo I

## Abstract

Cancer is one of the most serious diseases threatening human health, so it is particularly important to develop effective tumor-targeting drugs. As the first CDK4/6 inhibitor, palbociclib effectively inhibits tumor proliferation by blocking the cell cycle to the G1 phase. 10-HCPT is a Topo I inhibitor; however, its clinical application has been greatly limited due to its high toxicity. Based on the successful development of double target inhibitors, three novel palbociclib derivatives (*HP-1*, *HP-2*, and *HP-3*) were designed and synthesized from Palbociclib and 10-HCPT, and their biological activities were investigated. At first, the possible binding sites of the three compounds to Topo I and CDK4/6 were predicted by molecular docking. Then, we evaluated the anti-proliferative effects of the three palbociclib derivatives. In general, human lung cancer cells were more sensitive to HP-1, HP-2, and HP-3, especially NCI-H460. In addition, cell cycle arrest and apoptosis induction were investigated by flow cytometry. The three palbociclib derivatives, especially HP-1, had obvious cell cycle arrest phenomenon on NCI-H460 cells and induced apoptosis of NCI-H460 cells significantly. In the end, it was proved that these three drugs had obvious cyclin-dependent kinase inhibitory activities. In short, all the data showed that *HP-1*, *HP-2*, and *HP-3* could play anti-cancer roles by acting on dual targets and had the characteristics of high efficiencies and low toxicities, which opened up a new idea for the study of palbociclib derivatives.

## 1 Introduction

At present, chemotherapy is still the first choice for cancer patients in clinical application (1), while chemotherapy, surgery, radiotherapy, biological therapy, and other adjuvant therapies are common treatment methods for cancer treatment (2). Palbociclib is the first CDK4/6 inhibitor in the world (3) and approved by the Food and Drug Administration (FDA) in 2015. By inhibiting the phosphorylation of Rb protein, this drug leads to the disintegration of Rb protein binding transcription factor E2F (4), thus impeding the transcription of downstream molecules and leading to cell cycle arrest in the G1 phase (4). Therefore, in recent years, in the research and development of anti-tumor drugs, CDK4/6 has become a hot spot to achieve the purpose of anti-tumor by regulating the protein expression level in the signaling pathway (5).

Disordered cell cycle is a typical feature of tumor cells (6). Cyclin-dependent kinases (CDKs) play a crucial role in the regulation of cell cycle (7) and are specific therapeutic targets that interfere with the division and proliferation of tumor cells (8). At present, the clinical use of CDK4/6 inhibitor, palbociclib, also has many side effects. The most common side effect is neutropenia (9). Other common adverse reactions were infection, fatigue, nausea, and stomatitis in order (10). Therefore, it is necessary to develop palbociclib derivatives.

In the past studies, researchers focused on the study of palbociclib combination, and there are few reports on the derivatives of palbociclib. Based on the study of the mechanism of action of palbociclib, we modified it with 10-hydroxycamptothecin(10-HCPT). Camptothecin compounds are the only type of topoisomerase inhibitors that have been applied clinically and were first isolated from *Camptotheca acuminata* by Wall and Wani (1996) (11). Subsequently, it was found that HCPT had the highest activity among camptothecin compounds (12). Later in 1985, the mechanism of action was described as an inhibitor of the deoxyribonucleic acid topoisomerase I (Topo I) enzyme, defined by Xiang et al. (13).

Based on the successful case of benol ester (14), we proposed to use 10-HCPT and palbociclib as raw materials to provide three novel compounds (*HP-1*, *HP-2*, and *HP-3*) and investigated their biological activities. The results suggest that three novel compounds synthesized in this study may be a potential therapeutic strategy for the future.

## 2 Materials and methods

Palbociclib, 10-HCPT, 1-(3-dimethylaminopropyl)-3-ethylcarbodiimide hydrochloride (EDC), and 4-dimethylaminopyridine (DMAP) were purchased from Aladdin Chemical Reagent Company. Glutaric anhydride, succinic anhydride, and maleic anhydride were purchased from Budweiser Chemical Reagent Company. Anhydrous ethanol, methanol, dichloromethane, petroleum ether, ethyl acetate, potassium hydroxide, sodium hydroxide, sodium chloride, anhydrous sodium sulfate, and *N,N*-dimethylformamide (DMF) were purchased from Nanjing Wanqing Chemical Reagent Co., Ltd. L15 medium, 1640 medium, Dulbecco’s modified Eagle’s medium (DMEM), F12K medium, phosphate-buffered saline (PBS), 0.05% trypsin EDTA, and fetal bovine serum (FBS) were purchased from Gibco (USA). 3-(4,5-Dimethylthiazol-2-yl)-2,5 diphenyl tetrazolium bromide (MTT) cell proliferation and cytotoxicity test kit and annexin V-FITC/PI cell apoptosis test kit were purchased from Beyotime Biotechnology Co., Ltd. (China). Bicinchoninic acid (BCA) protein quantitative kit and protein maker were purchased from Thermo (USA). Polyvinylidene fluoride (PVDF) Western blotting membranes were purchased from Biosharp (China). The enhanced chemiluminescence reagent was purchased from Cell Signaling Company (USA). Dimethyl sulfoxide (DMSO) was purchased from Nanjing Chemical Reagent Co., Ltd. (China). The antibodies used in the experiment were purchased from Wanlei Biotechnology Co., Ltd. (China).

### 2.1 Molecular docking

Crystal structures of protein CDK4, CDK6, and Topo I were retrieved from Protein Data Bank (PDB code: 1RR8,2W99,5L2I). 3D structures of *HP-1*, *HP-2*, and *HP-3* were prepared and geometrically optimized by Discovery Studio 3.1. Standard ligand molecule preparation, such as addition of hydrogen atoms, bond lengths, and bond angle correction, co-crystallized water molecules removal, and energy minimization, was performed before docking. Finally, the molecular docking software was used to simulate the docking of the domains of the target proteins Topo I, CDK4, and CDK6 kinases with compounds *HP-1*, *HP-2*, *HP-3*, HCPT, and palbociclib, respectively, and the corresponding CDocker interaction energy calculation results were obtained.

### 2.2 Synthesis of *HP-1, HP-2*, and *HP-3*

**Figure 1:**
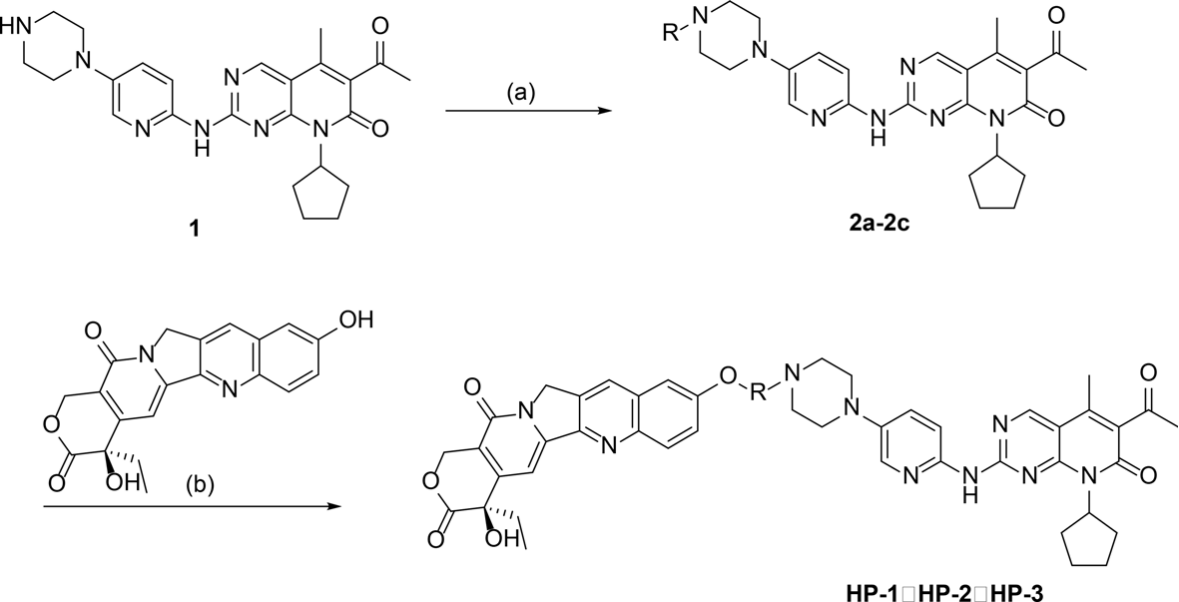
The synthetic routes of palbociclib 10-HCPT ester derivatives (***HP-1***, ***HP-2***, and ***HP-3***) [reagents and conditions: **(A)** DMF, anhydride, 70°C, 24 h; **(B)** EDCI, DMAP, CH_2_Cl_2_, 4 h].

The anhydride (0.25 mmol) and palbociclib (0.25 mmol) were dissolved in 50 ml DMF with stirring at 70°C for 12 h. The obtained organic layer was washed with dilute hydrochloric acid and saturated salt water in turn, and concentrated. The solids were recrystallized in hexane, filtered to obtain the crystallization products, and dried in a vacuum-drying oven. The products were palbociclib derivatives (**2a–2c**), and the reaction route is shown in **Figure 1**.

**Figure 2:**
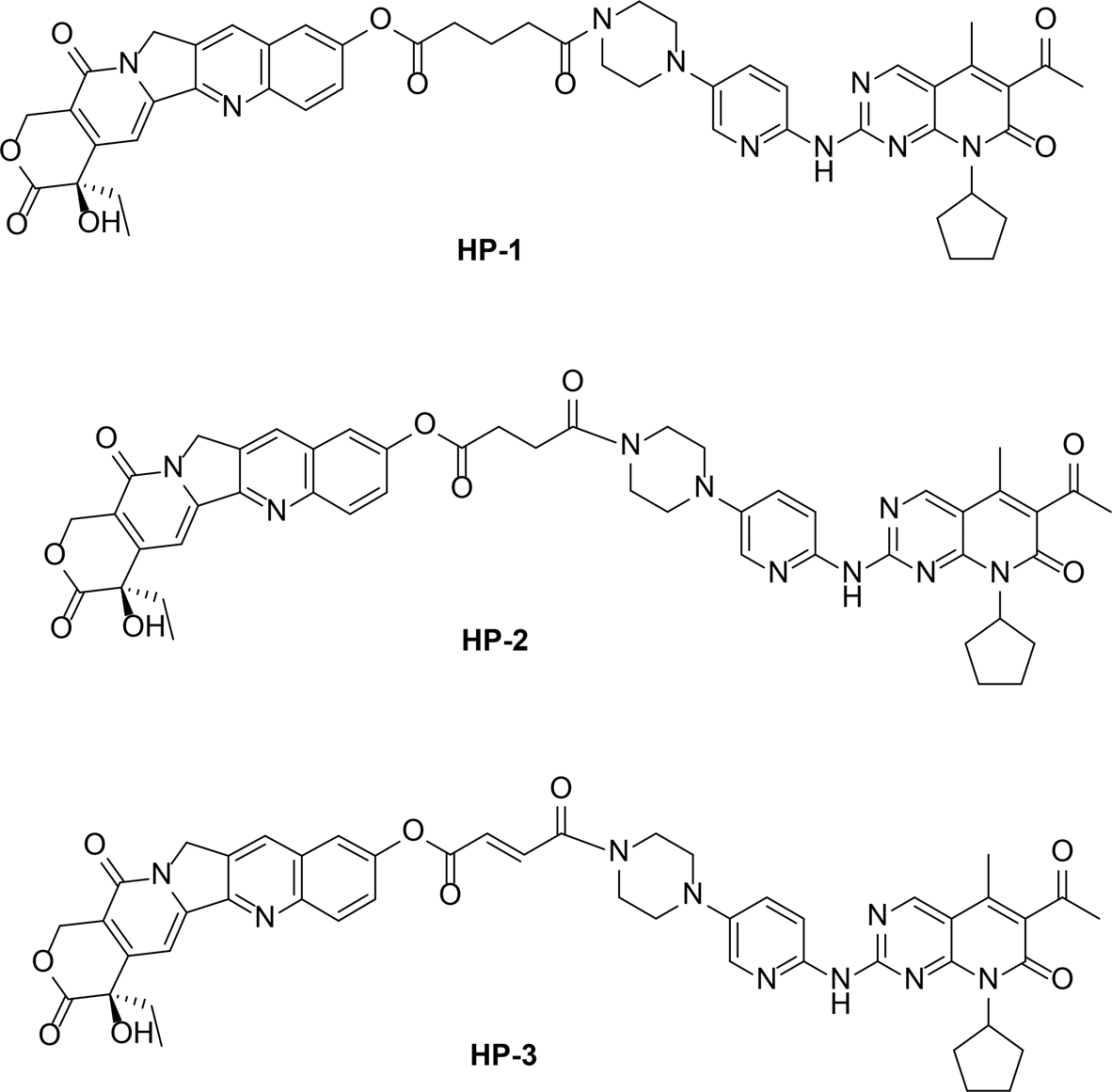
Chemical structures of ***HP-1*, *HP-2***, and ***HP-3***.

10-HCPT (0.175 mmol), EDCI (0.354 mmol), and DMAP (0.175 mmol) were added to a solution of **2a-2c** (0.175 mmol) in 15 ml of CH_2_Cl_2_ with stirring at 0°C for 4 h. The products were three novel palbociclib derivatives (HP-1, HP-2, and HP-3) (**Figure 2**), and the reaction route is shown in **Figure 1**.

#### 2.2.1 ^1^H-NMR and MS parameters of *HP-1*, *HP-2*, and *HP-3*

(R)-4-ethyl-4-hydroxy-3,14-dioxo-3,4,12,14-tetrahydro-1H-pyrano[3’,4’:6,7] indolizino[1,2-b]quinolin-9-yl5-(4-(6-((6-acetyl-8-cyclopentyl-5-methyl-7-oxo-7,8-dihydropyrido[2,3-d]pyrimidin-2-yl)amino)pyridin-3-yl)piperazin-1-yl)-5-oxopentanoate(*HP-1*)

Yellow powders, yield 75%. Mp: 44.5-46.4°C.^1^H NMR (400 MHz, CDCl_3_) *δ* 9.47 (s, 1H), 8.34 (s, 1H), 8.26 (d, *J* = 2.2 Hz, 1H), 8.22 (d, *J* = 2.1 Hz, 1H), 7.97 (d, *J* = 8.1 Hz, 1H), 7.65 (t, *J* = 2.1 Hz, 1H), 7.38 (dd, *J* = 8.3, 1.9 Hz, 1H), 7.27 (t, *J* = 0.9 Hz, 1H), 7.08 (dd, *J* = 8.3, 2.3 Hz, 1H), 6.92 (d, *J* = 8.3 Hz, 1H), 5.23 (dd, *J* = 12.5, 1.1 Hz, 1H), 4.92 (s, 2H), 4.77–4.70 (m, 2H), 4.45 (p, *J* = 5.7 Hz, 1H), 3.66 (dd, *J* = 6.2, 3.5 Hz, 2H), 3.62 (dd, *J* = 6.2, 3.5 Hz, 2H), 3.18 (ddd, *J* = 6.4, 4.9, 3.6 Hz, 4H), 2.73 (s, 2H), 2.61–2.51 (m, 2H), 2.40–2.33 (m, 1H), 2.36 (s, 4H), 2.19 (dddd, *J* = 11.1, 5.7, 4.4, 2.1 Hz, 4H), 2.01–1.92 (m, 1H), 1.94–1.85 (m, 2H), 1.85–1.74 (m, 1H), 1.77–1.65 (m, 2H), 1.69–1.57 (m, 2H), 0.97 (t, *J* = 8.0 Hz, 3H) (**Supplementary Figure S1**). ESI-MS: calcd for C_49_H_49_N_9_O_9_ [M+H]^+^ *m/z*, 907.99; found, 907.95 (**Supplementary Figure S4**). Anal. Calcd for C_49_H_49_N_9_O_9_: C, 64.82; H, 5.44; N, 13.88; O, 15.86. Found: C, 64.79; H, 5.40; N, 13.90; O, 15.87.

(R)-4-ethyl-4-hydroxy-3,14-dioxo-3,4,12,14-tetrahydro-1H-pyrano[3’,4’:6,7] indolizino[1,2-b]quinolin-9-yl4-(4-(6-((6-acetyl-8-cyclopentyl-5-methyl-7-oxo-7,8-dihydropyrido[2,3-d]pyrimidin-2-yl)amino)pyridin-3-yl)piperazin-1-yl)-4-oxobutanoate (*HP-2*)

Yellow powders, yield 83%. Mp: 48.9-50.5°C. ^1^H NMR (400 MHz, CDCl_3_) *δ* 9.47 (s, 1H), 8.34 (s, 1H), 8.26 (d, *J* = 2.2 Hz, 1H), 8.22 (d, *J* = 2.4 Hz, 1H), 7.97 (d, *J* = 8.2 Hz, 1H), 7.63 (t, *J* = 2.1 Hz, 1H), 7.38 (dd, *J* = 8.3, 1.9 Hz, 1H), 7.27 (t, *J* = 0.9 Hz, 1H), 7.08 (dd, *J* = 8.3, 2.3 Hz, 1H), 6.92 (d, *J* = 8.3 Hz, 1H), 5.23 (dd, *J* = 12.5, 1.1 Hz, 1H), 4.92 (s, 2H), 4.77–4.70 (m, 2H), 4.45 (p, *J* = 5.7 Hz, 1H), 3.66 (dd, *J* = 6.2, 3.5 Hz, 2H), 3.62 (dd, *J* = 6.2, 3.5 Hz, 2H), 3.18 (td, *J* = 6.3, 3.5 Hz, 4H), 2.80–2.71 (m, 5H), 2.71–2.58 (m, 2H), 2.36 (s, 3H), 2.19 (dddd, *J* = 11.0, 5.6, 4.3, 2.1 Hz, 4H), 1.96 (dq, *J* = 13.9, 8.1 Hz, 1H), 1.85–1.76 (m, 1H), 1.79–1.68 (m, 2H), 1.71–1.57 (m, 3H), 0.97 (t, *J* = 8.0 Hz, 3H) (**Supplementary Figure S2**).ESI-MS: calcd for C_48_H_47_N_9_O_9_ [M+H]^+^ *m/z*,893.96; found, 893.93 (**Supplementary Figure S5**). Anal. Calcd for C_48_H_47_N_9_O_9_: C, 64.49; H, 5.30; N, 14.10; O, 16.11. Found: C, 64.44; H, 5.26; N, 14.03; O, 16.09.

(R)-4-ethyl-4-hydroxy-3,14-dioxo-3,4,12,14-tetrahydro-1H-pyrano[3’,4’:6,7] indolizino[1,2-b]quinolin-9-yl(E)-4-(4-(6-((6-acetyl-8-cyclopentyl-5-methyl-7-oxo-7,8-dihydropyrido[2,3-d]pyrimidin-2-yl)amino)pyridin-3-yl)piperazin-1-yl)-4-oxobut-2-enoate (*HP-3*)

Yellow powders, yield 77%. Mp: 45.7–47.3°C. ^1^H NMR (400 MHz, CDCl_3_) *δ* 9.47 (s, 1H), 8.34 (s, 1H), 8.26 (d, *J* = 2.2 Hz, 1H), 8.20 (d, *J* = 2.1 Hz, 1H), 7.95 (d, *J* = 8.4 Hz, 1H), 7.62 (t, J = 2.1 Hz, 1H), 7.42 (dd, *J* = 8.3, 1.9 Hz, 1H), 7.27 (t, *J* = 0.9 Hz, 1H), 7.08 (dd, *J* = 8.3, 2.3 Hz, 1H), 6.92 (d, *J* = 8.3 Hz, 1H), 6.86 (d, *J* = 16.7 Hz, 1H), 6.64 (d, *J* = 16.9 Hz, 1H), 5.23 (dd, *J* = 12.5, 1.1 Hz, 1H), 4.92 (s, 2H), 4.77–4.70 (m, 2H), 4.45 (p, *J* = 5.7 Hz, 1H), 3.82 (dd, *J* = 6.3, 3.6 Hz, 2H), 3.76 (dd, *J* = 6.3, 3.6 Hz, 2H), 3.28 (ddd, *J* = 6.2, 3.5, 1.3 Hz, 4H), 2.73 (s, 2H), 2.36 (s, 2H), 2.25–2.12 (m, 4H), 1.96 (dq, *J* = 13.9, 8.1 Hz, 1H), 1.85–1.72 (m, 1H), 1.77–1.64 (m, 2H), 1.69–1.57 (m, 2H), 0.97 (t, *J* = 8.0 Hz, 3H) (**Supplementary Figure S3**). ESI-MS: calcd for C_48_H_45_N_9_O_9_ [M+H]^+^ *m/z*, 891.94; found, 891.97 (**Supplementary Figure S6**). Anal. calcd for C_48_H_45_N_9_O_9_: C, 64.64; H, 5.09; N, 14.13; O, 16.14. Found: C, 64.61; H, 5.01; N, 14.05; O, 16.1.

### 2.3 Cell cultures

Human lung cancer cells (NCI-H460, NCI-H1299, and A549), human breast cancer cells (MDA-MB-231, MDA-MB-453, and MCF-7), human liver cancer cells (Huh-7 and HepG2), human colon cancer cells (coca-2, HT-29, and HCT116), human cervical cancer cells (HeLa), human normal lung cells (BEAS-2B), and human normal breast cancer cells (MCF-10) were from the State Key Laboratory of Pharmaceutical Biotechnology, Nanjing University. NCI-H460 were cultured in 1640 medium containing 10% fetal bovine serum (FBS). MDA-MB-231 were cultured in L15 medium containing 10% FBS without CO_2_, and other cell lines were cultured in DMEM medium containing 10% FBS CO_2_ in the incubator.

### 2.4 *In vitro* cytotoxicity

MTT assay was used to identify 14 different cell lines (human lung cancer cells NCI-H460, NCI-H1299, and A549; human breast cancer cells MDA-MB-231, MDA-MB-453, and MCF-7; human liver cancer cells Huh-7 and HepG2; human colon cancer cells coca-2, HT-29, and HCT116; and human cervical cancer cells HeLa) and 2 kinds of normal cell lines (human normal lung epithelial cells BEAS-2B, human normal breast epithelial cells MCF10-A) for quantitative analysis (15). In addition, 10-HCPT and palbociclib were used as positive control.

Cells were harvested during the logarithmic growth phase and seeded into 96-well plates at a density of 2.0×10^3^ cells/well. After being incubated for 24 h, the cells were treated with different concentrations of various culture programs for 48 h. Afterwards, 20 μl of MTT solution (5 mg/ml) was added to each well, and the cells were incubated in a 5% CO_2_ atmosphere at 37°C for another 4 h. Then, the supernatant was discarded, and the formazan crystals were dissolved in 150 μl DMSO. Subsequently, the absorbance was measured at 570 nm *via* a Tecan microplate reader. The concentration that caused 50% inhibition of cell viability (IC_50_) values was calculated by GraphPad Software. Triplicate experiments were performed in parallel for each concentration, and the results are shown as the mean ± SD. The inhibitory ratio was calculated using the following formula: inhibitory ratio (%) = [1−(mean absorbance of experiment group)/(mean absorbance of control group)] × 100.

### 2.5 Cell cycle analysis

The cell cycle distribution was measured by flow cytometry. NCI-H460 cells were harvested in logarithmic growth phase and seeded into six-well plates at a density of 5.0 × 10^3^ cells/well. After being incubated for 24 h, the cells were harvested with 0.25% Trypsin after exposed to various culture programs. The experiment was triply replicated. Then, the cell was fixed in 500 μl 70% ethanol overnight at 4°C. Subsequently, 100 μl RNase A and 400 μl PI were added to the cell suspension, and cells were incubated for 30 min at 4°C in the dark.

### 2.6 Western blot analysis

NCI-H460 cells were harvested in logarithmic growth phase and seeded into six-well plates at a density of 5.0 × 10^3^ cells/well. After being incubated for 48 h, the cells were harvested with 0.25% Trypsin after exposed to various concentrations of HP-1 (0, 0.5, 1, 2 μM), 10-HCPT (2 μM), and pabocini (2 μM) for 24 h.Then, precooled radio-immunoprecipitation assay (RIPA) lysates [10 mM Tris–HCl, 1 mM EDTA, 1% sodium dodecyl sulfate (SDS), 1 mM dithiothreitol (DTT), 0.1 mM phenylmethylsulfonyl fluoride (PMSF), protease inhibitors, 1% Nonidet P-40, pH 8] were added to them and placed on ice to lyse the cells for 30 min. After centrifugation at 10,000 rpm and 4°C for 10 min, the supernatant was collected to obtain soluble proteins. Lysates were centrifuged at 10,000 rpm for 20 min at 4°C, and the supernatant was collected.

The absorbance value was measured at 562 nm using a microplate analyzer to calculate the protein content. An equal amount of protein was separated by 10% SDS–polyacrylamide gel electrophoresis (SDS-PAGE) gels and transferred onto PVDF membrane. The membranes were blocked with 5% SPM in Tris-buffered saline buffer containing Tween-20 (TBST) for 1 h and then incubated with specific primary antibodies diluted in 5% SPM buffer overnight at 4°C. After being washed with TBST buffer for three times every 5 min, the membranes were incubated with secondary antibodies diluted in TBST buffer for 1 h at room temperature. Finally, the membrane was placed on the membrane carrier plate, and the bands were detected with enhanced chemiluminescence reagent to indicate the protein expression.

### 2.7 Annexin V/PI staining

NCI-H460 cells were harvested in logarithmic growth phase and seeded into six-well plates at a density of 5.0 × 10^3^ cells/well. After being incubated for 24 h, the cells were harvested with 0.25% Trypsin after exposed to various culture programs, washed with PBS, and re-suspended in 500 μl binding buffer. The experiment was triply replicated. Then, the cells were stained by adding to 5 μl Annexin V-FITC and 5 μl PI for15 min at 25°C in the dark. Finally, the cells were detected and analyzed using a flow cytometer with FlowJo 7.6.1.

## 3 Results and discussions

### 3.1 Molecular docking

The binding energy and mode of interaction between target protein and molecule ligand were determined by molecular docking simulation. **Table 1** shows the results.

**Table 1:** Docking simulation results of ***HP-1***, ***HP-2***, and ***HP-3***, 10-HCPT, and palbociclib with 1RR8, 2W99, and 5L2I.

Compounds	-CDocker interaction energy△G(kcal/mol)		
	Topo I (1RR8)	CDK4 (2W99)	CDK6 (5L2I)
***HP-1***	99.9908	56.5927	67.7225
***HP-2***	92.9532	53.6718	65.2603
***HP-3***	82.4775	52.1803	64.6216
**10-HCPT**	44.4342	–	–
**Palbociclib**	–	40.5647	64.7078

According to the results of computer virtual screening, it can be concluded that the absolute value of binding energy of *HP-1*, *HP-2*, and *HP-3* and target proteins Topo I (1RR8), CDK4 (2W99), and CDK6 (5L2I) is greater than that of 10-HCPT and palbociclib. In other words, *HP-1*, *HP-2*, and *HP-3* can be better bound to these two target proteins. Therefore, we infer that these three compounds inhibit tumor cell proliferation and block tumor cell cycle progression by acting simultaneously on Topo I and CDK4/6. In addition, among the three molecules designed, *HP-1* had the highest absolute binding energy with Topo I, CDK4, and CDK6 targets, which was 99.9908, 56.5927, and 67.7225 kcal/mol, respectively. *HP-1* has the highest theoretical activity value.

In order to describe the interaction between the compound and the three target proteins in a better way, *HP-1* with the largest absolute binding energy was selected as an example in this paper.

*HP-1* forms a hydrogen bond with Lys374 in the cavity of Topo I protein (**Supplementary Figure S7A**), which makes the compound bind to Topo I target protein more closely, which is also the main reason why the binding energy of *HP-1* to 1RR8 target protein is much higher than that of 10-HCPT. In addition, the introduction of palbociclib allowed the small molecule *HP-1* to be linked to the Topo I protein through more chemical bonds. These structures interact with GLu356, Lys425, DC112, Da113, and Tyr426 amino acid residues in the cavity to form π-cation bonds, π-anion bonds, and π-alkyl bonds.

10-HCPT cannot only form a hydrogen bond with Gly335 and Thr718 in the cavity structure of Topo I protein to stably bind to Topo I (**Supplementary Figure S7C**) but also form other weak interactions with Arg364, Asp533, Arg364, DG12, and other proteins, such as π-cation bond and π-anion bond, and π-alkyl.

*HP-1* is embedded in the cavity structure of Topo I protein in the form of a hemicloop (**Supplementary Figures S7B, D**) and is tightly linked to Topo I protein through multiple interactions. Therefore, compared with 10-HCPT, the binding of *HP-1* to the target protein is more stable.

Palbociclib mainly binds to CDK4 protein through a strong hydrogen bond and several weak π-cation and π-alkyl bonds (**Supplementary Figure S8**). The introduction of 10-HCPT structure in *HP-1* molecule brings an additional hydrogen bond and three π-anion bonds to the compound, so the binding of *HP-1* to CDK4 is tighter than that of palbociclib.

The binding of palbociclib to CDK6 protein is mainly through one hydrogen bond and multiple π-alkyl bonds (**Supplementary Figure S9**). The effect of *HP-1* and CDK6 is not only related to π-alkyl bond but also to hydrogen bond. Because the hydrogen bond energy is higher, the binding energy of *HP-1* and CDK6 is higher than that of palbociclib.

The results of the molecular docking suggested that the synthesized compounds exhibited good affinity to the target protein. This also indicated that *HP-1*, *HP-2*, and *HP-3* may inhibit the growth of tumor cells through their interactions with Topo I, CDK4, and CDK6.

### 3.2 *In vitro* cytotoxicity

We investigated the inhibitory effect of drugs on different human cancer cells by MTT assay. After treatment with various concentrations of five drugs for 48 h, the result of inhibitory effects is shown in **Table 2**.

**Table 2:** IC_50_ value of compounds *HP-1*, *HP-2*, and *HP-3*.

CompoundsCells	*HP-1*	*HP-2*	*HP-3*	10-HPCT	Palbociclib
**NCI-H460**	0.96 ± 0.01**^/##^	1.32 ± 0.01*^/##^	2.08 ± 0.25^#^	1.96 ± 0.01	2.95 ± 0.11
**NCI-H1299**	2.29 ± 0.02*^/##^	3.35 ± 0.01^##^	4.35 ± 0.13*^/#^	3.39 ± 0.16	5.09 ± 0.24
**A549**	3.41 ± 0.22*^/##^	5.24 ± 0.37*^/#^	5.52 ± 0.11*^/#^	4.14 ± 0.03	6.16 ± 0.19
**BEAS-2B**	36.38 ± 0.43**	34.75 ± 0.44**	39.98 ± 0.16**	7.05 ± 0.52	38.75 ± 0.59
**MDA-MB-231**	15.30 ± 2.51^#^	16.54 ± 0.82^#^	27.26 ± 1.43**^/##^	15.53 ± 1.07	11.67 ± 1.75
**MDA-MB-453**	5.28 ± 0.81	6.38 ± 0.97^#^	8.31 ± 0.52*^/##^	5.73 ± 0.62	5.15 ± 1.04
**MCF-7**	10.31 ± 1.68*^/#^	16.73 ± 0.59*^/##^	21.45 ± 1.27**^/##^	13.21 ± 0.31	8.73 ± 0.33
**MCF10-A**	32.42 ± 1.33**^/#^	31.38 ± 0.23**^/##^	37.28 ± 1.66**	20.26 ± 0.59	39.69 ± 0.57
**Huh-7**	9.48 ± 1.59^##^	16.52 ± 1.76**^/##^	33.67 ± 1.03**	7.69 ± 0.84	32.13 ± 1.93
**HepG2**	2.57 ± 0.42^##^	8.11 ± 1.34**	6.84 ± 0.91**	2.45 ± 0.27	7.55 ± 1.49
**Coca-2**	15.16 ± 0.54^##^	18.82 ± 0.46*^/##^	17.12 ± 0.28*^/##^	13.41 ± 0.34	35.88 ± 0.41
**HT-29**	12.39 ± 1.62**^/#^	11.59 ± 0.57**^/##^	15.36 ± 0.42**	4.61 ± 0.19	16.77 ± 0.39
**HCT116**	17.07 ± 0.31**^/##^	19.64 ± 1.21**^/##^	23.18 ± 0.49**^/##^	10.41 ± 0.70	31.64 ± 1.21
**Hela**	5.46 ± 0.08^##^	7.82 ± 2.34^##^	14.89 ± 0.73**^/##^	6.54 ± 0.07	21.35 ± 0.13

Data shown are the mean ± SD ( $\bar{x}$ ± SD, µM) of three independent experiments (^*^p<0.05, ^**^p<0.01, compared to HCPT; ^#^ p<0.05, ^##^ p<0.01, compared to albociclib).

For lung cancer cells, *HP-1*, *HP-2*, and *HP-3* showed anti-proliferation activities, and NCI-H460 cells were more sensitive to the three novel palbociclib derivatives. In addition, the cytotoxicity of *HP-1* (IC_50_ = 36.38 μM), *HP-2* (IC_50_ = 34.75 μM), and *HP-3* (IC_50_ = 39.98 μM) to human normal lung epithelial cells BEAS-2B was slightly higher than that of palbociclib (IC_50_ = 38.75 μM) but much lower than that of 10-hydroxycamptocampin (IC_50_ = 7.05 μM).

Similarly for human breast cancer cells, the inhibitory effect of *HP-1* and *HP-2* was better than that of 10-hydroxycamptocampin. Furthermore, the cytotoxicity of *HP-1* (IC_50_ = 32.42 μM), *HP-2* (IC_50_ = 31.38 μM), and *HP-3* (IC_50_ = 37.28 μM) to normal mammary cell MCF-10A was much lower than that of 10-HCPT (IC_50 =_ 20.26 μM) but slightly higher than that of palbociclib (IC_50_ = 39.69 μM).

In general, human lung cancer cells were more sensitive to *HP-1*, *HP-2*, and *HP-3*, especially NCI-H460. It is worth emphasizing that their inhibitory effect on NCI-H460 cells was better than that of 10-HCPT and palbociclib, so NCI-H460 cells were selected for subsequent studies.

### 3.3 Cell cycle arrest

**Figure 3:**
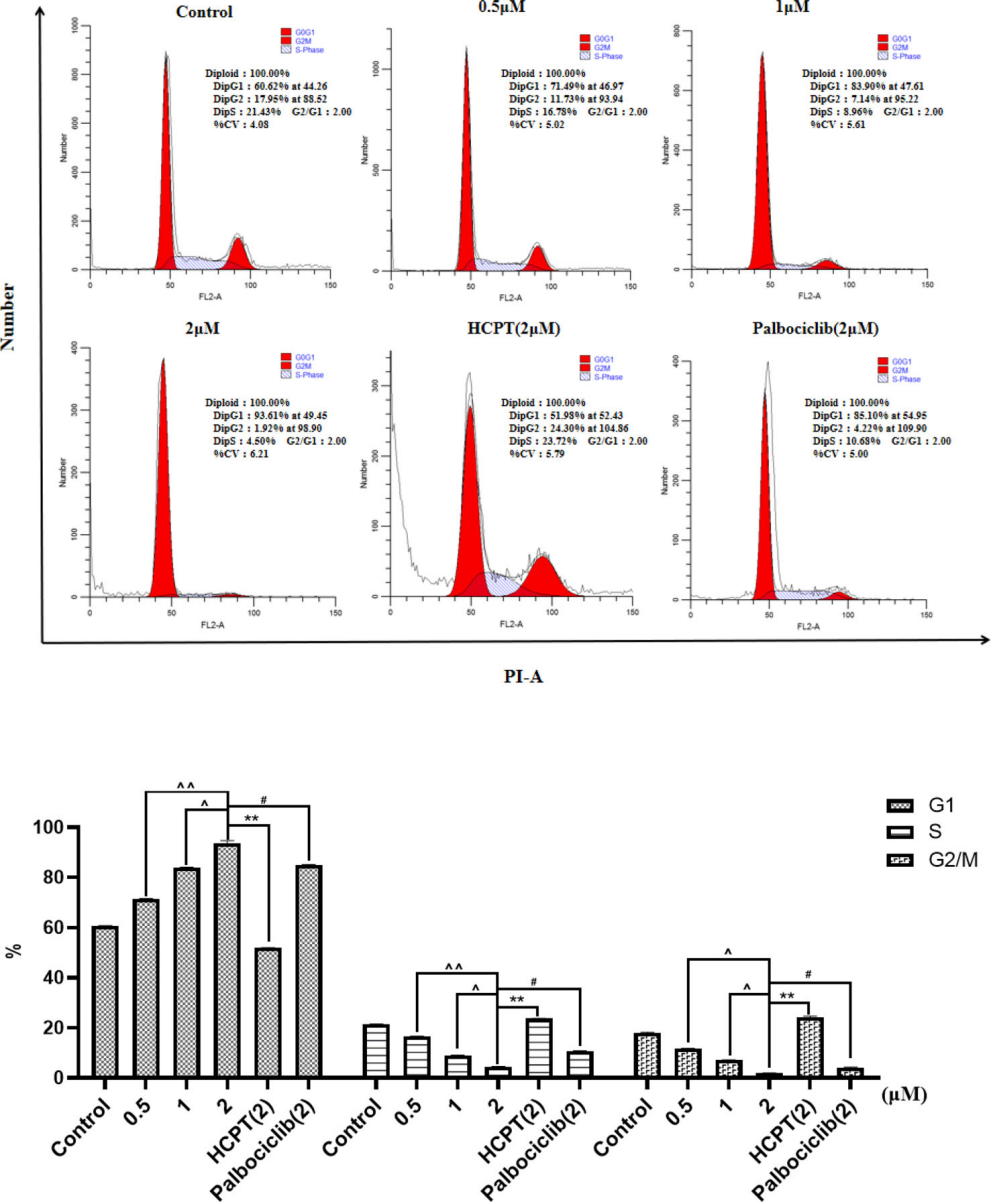
Effect of ***HP-1*** on the cell cycle of NCI-H460 cells in concentration dependence. Data shown are the mean ± SD of three independent experiments [^**^ *p*< 0.01, compared to HCPT; ^#^ *p*<0.05, compared to palbociclib; ***^^^****p*<0.05, ***^^^^****p*<0.01, compared to HP-1 (2 μM)].

**Figure 3** and **Supplementary Figures S10, S11** show the concentration-dependent cell cycle arrest effect of compounds *HP-1*, *HP-2*, and *HP-3* on NCI-H460 cells, respectively. The results indicated that with the increase in compound concentration, more NCI-H460 cells were arrested in the G1 phase, and *HP-1* works best. In addition, the cell cycle arrest effect of NCI-H460 cells treated with 2 μM *HP-1* in G1 phase was better than that of 10-hydroxycamptocampin and Palbociclib at the same concentration.

**Supplementary Figure S12** shows the results of cycle arrest of BEAS-2B cells by different concentrations of *HP-1*, with 10-HCPT and palbociclib as positive controls. The results showed that with the increase in *HP-1* concentration, the G1 arrest rate of BEAS-2B cells increased slightly from 67.14% to 74.63%, while at the same concentration, the G1 arrest rate of NCI-H460 cells induced by *HP-1* increased significantly from 60.62% to 93.61%. Therefore, *HP-1* has little effect on normal cells.

**Figure 4:**
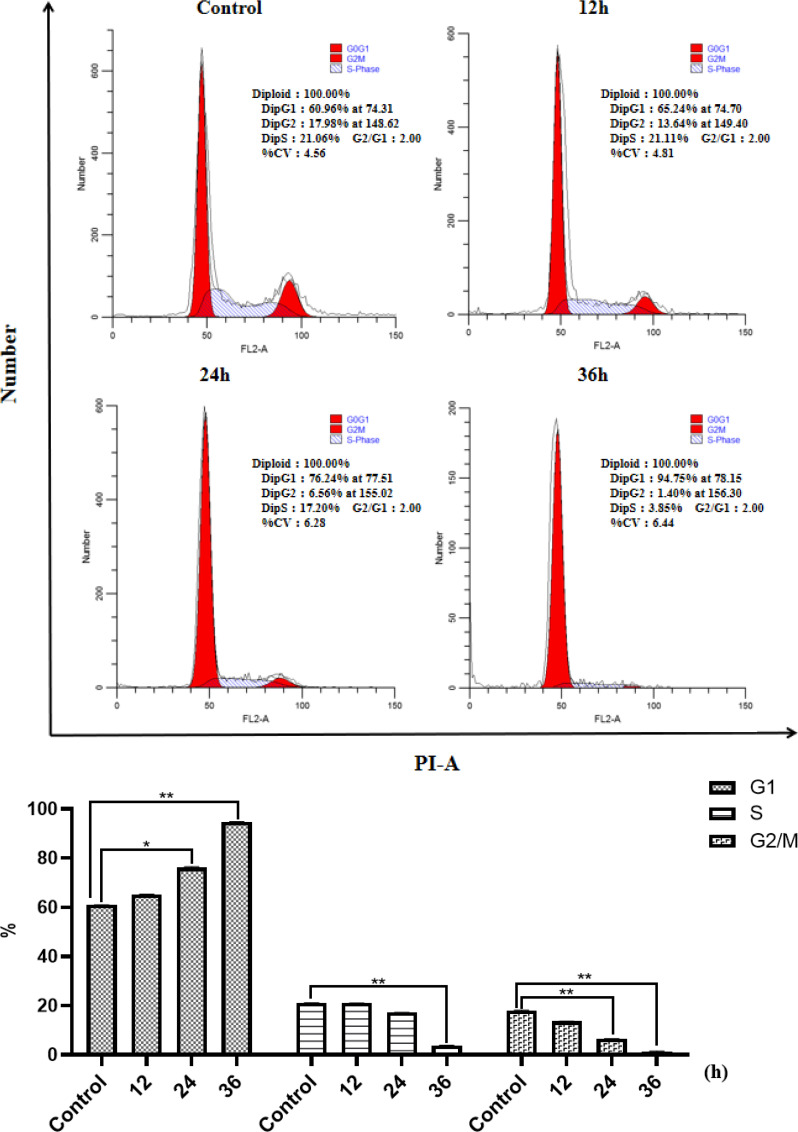
Effect of ***HP-1*** on the cell cycle of NCI-H460 cells in time dependence. Data shown are the mean ± SD of three independent experiments (^*^ *p*<0.05, ^**^ *p*<0.01, compared to negative control).

The cell cycle arrest results of NCI-H460 at 12, 24, and 36 h in the same concentration of *HP-1* are shown in **Figure 4**. It can be concluded that the proportion of NCI-H460 cell cycle arrest in G1 phase induced by *HP-1* increases significantly with time, from 60.96% to 94.75%.

### 3.4 Apoptotic cell

**Figure 5:**
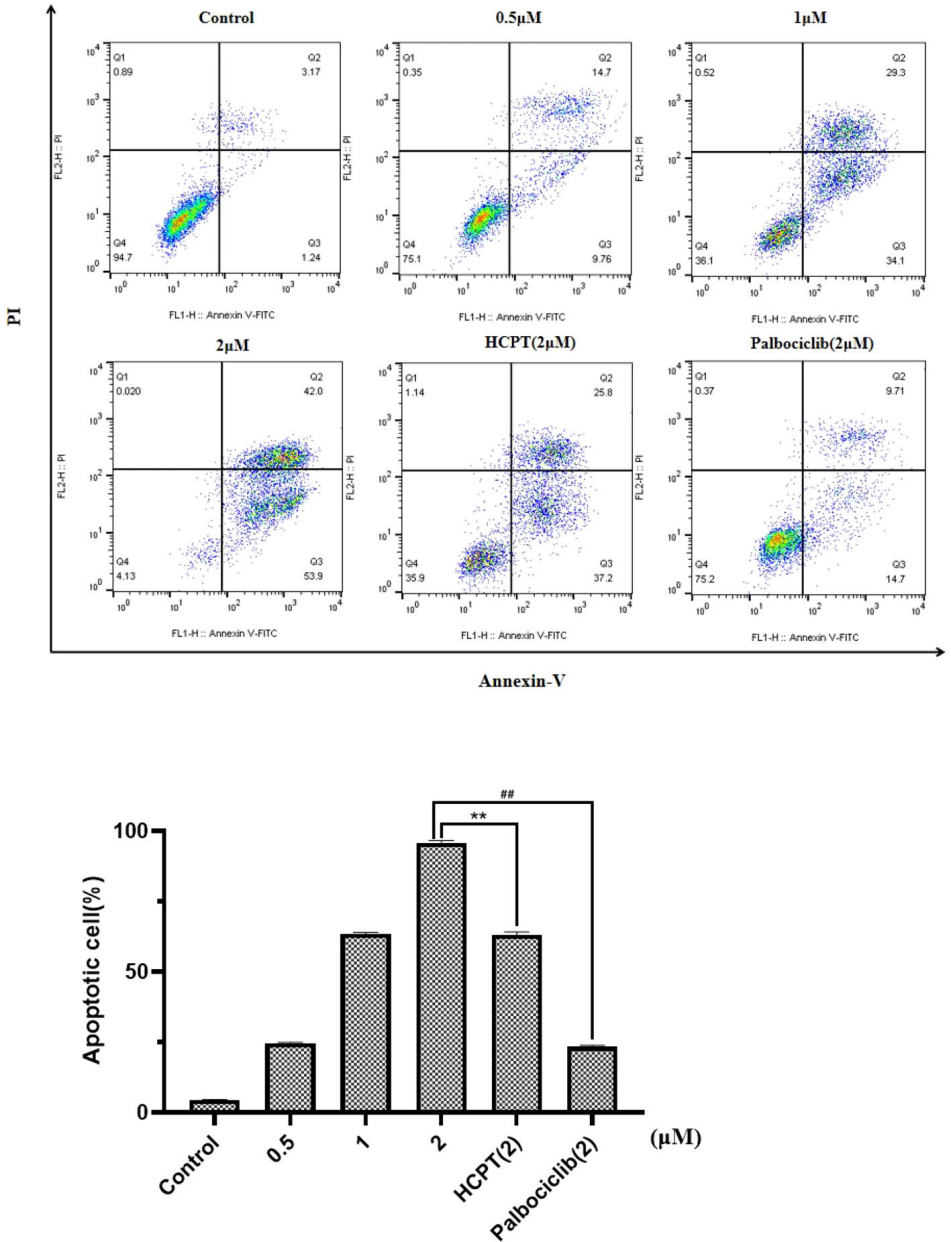
Effect of ***HP-1*** on the apoptosis of NCI-H460 cells in concentration dependence. Data shown are the mean ± SD of three independent experiments (^**^ *p*<0.01, compared to HCPT; ^##^ *p*<0.01, compared to palbociclib).

**Figure 5** and **Supplementary Figures S13, S14** show the effects of different concentrations of drugs on apoptotic cell. Compared with the negative control (0 μM), it can be concluded that different concentrations of *HP-1*, *HP-2*, and *HP-3* have different effects on the apoptosis of NCI-H460 cells. Taking HP-1 as an example, with the increase in *HP-1* concentration, the proportion of apoptotic cells increased gradually. The effect of *HP-1* on apoptosis was higher than that of palbociclib at the same concentration. Additionally, HP-2 and HP-3 induced NCI-H460 cell apoptosis by 4.37%–76.6% and 4.66%–64.9%, respectively.

In order to verify the effect of this compound on human normal lung cells BEAS-2B. *HP-1* was selected to treat BEAS-2B cells and compared with 10-HCPT and palbociclib. As shown in **Supplementary Figure S15**, with the increase in *HP-1* concentration, the apoptosis rate increased from 2.52% to 4.25%. Therefore, *HP-1* has little effect on normal cells.

**Figure 6:**
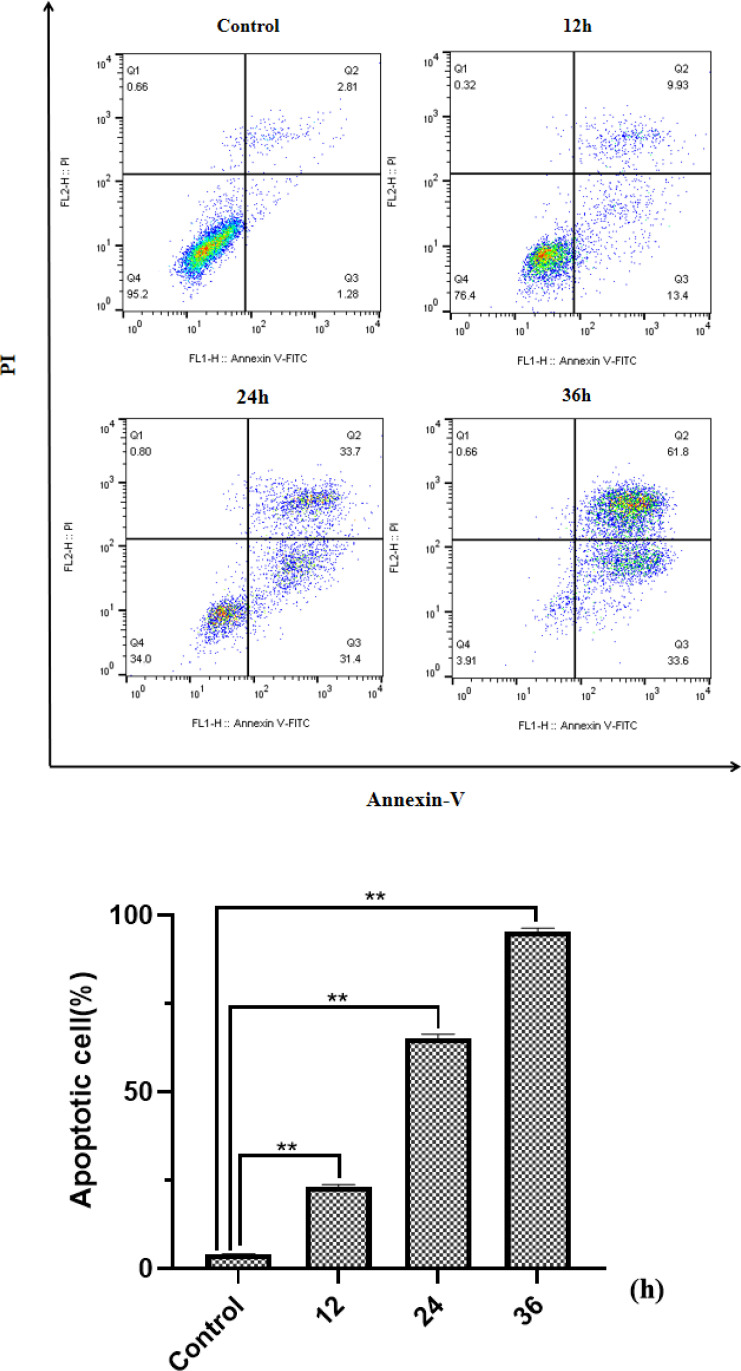
Effect of ***HP-1*** on the apoptosis of NCI-H460 cells in time dependence. Data shown are the mean ± SD of three independent experiments (^**^ *p*<0.01 compared to negative control).

Beside the drug concentration, the incubation time is also an important factor that affects the apoptosis. Under the same concentration of *HP-1* culture, the apoptosis rate of NCI-H460 cells at 12, 24, and 36 h is shown in **Figure 6**. The results show that the apoptotic rate of NCI-H460 cells induced by *HP-1* increased significantly with time, from 4.09% to 95.4%.

### 3.5 Analysis of CDK4/CDK6 signal transduction pathway-related proteins

**Figure 7:**
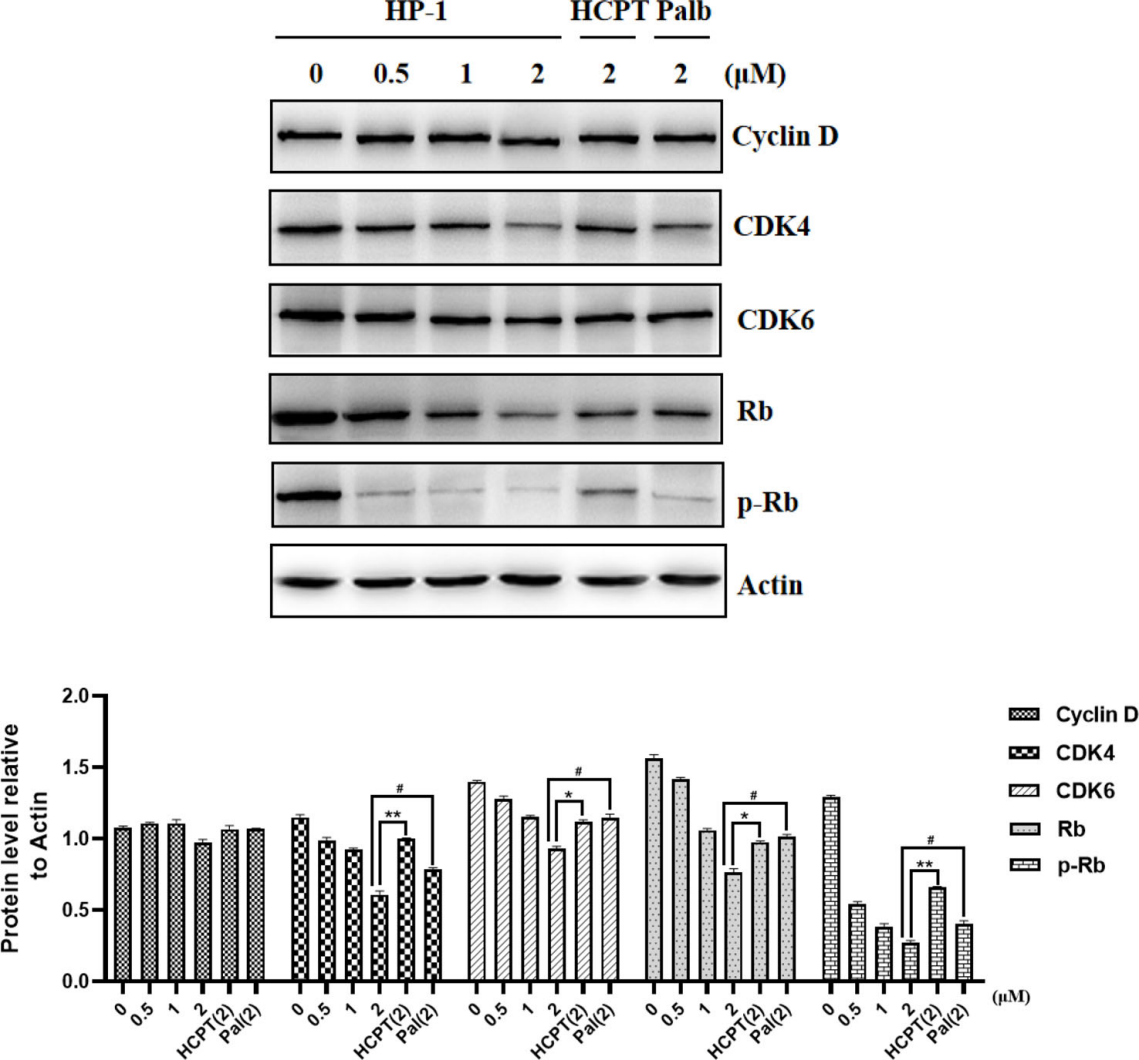
Effect of ***HP-1*** on Cyclin D-CDK4/6-Rb-2F pathway proteins of NCI-H460 cells. Data shown are the mean ± SEM of three independent experiments (^*^ *p*<0.05, ^**^ *p*<0.01, compared to HCPT; ^#^ *p*<0.05, compared to palbociclib).

Cyclin D is an important protein in cell cycle regulation. It can bind to CDK4/6 and phosphorylate retinoblastoma tumor suppressor protein (Rb), thereby inducing the cell cycle transition from G1 phase to S phase. From the above cell cycle arrest images, it can be concluded that the cells treated with *HP-1*, *HP-2*, and *HP-3* have obvious cycle arrest. In order to further elucidate their mechanism of action, Western blotting was used to detect the level of related proteins in the CyclInd-CDK4/6-Rb pathway, which regulates G1 phase. The experimental results are shown in **Figure 7**. Protein expressions of Cyclin D, CDK4, CDK6, Rb, and p-Rb are significantly decreased with the increase in *HP-1* concentration. This also indicates that *HP-1* does block cells in the G1 phase by Cyclin D-CDK4/6-Rb. In addition, the inhibition effect of *HP-1* on cyclins was better than that of palbociclib, which may be due to the enhanced effect of the introduction of 10-HPCT structure. Therefore, we suggest that *HP-1* is a potential G1 cell cycle inhibitor.

### 3.6 Analysis of apoptotic protein

The mechanism of *HP-1*-inducing apoptosis in cancer cells was explored. Considering that the way of apoptosis induced by 10-HCPT may be related to the PI3K/AKT pathway, we detected the effect of *HP-1* on the expression of PI3K/AKT pathway-related proteins in NCI-H460 cells.

**Figure 8:**
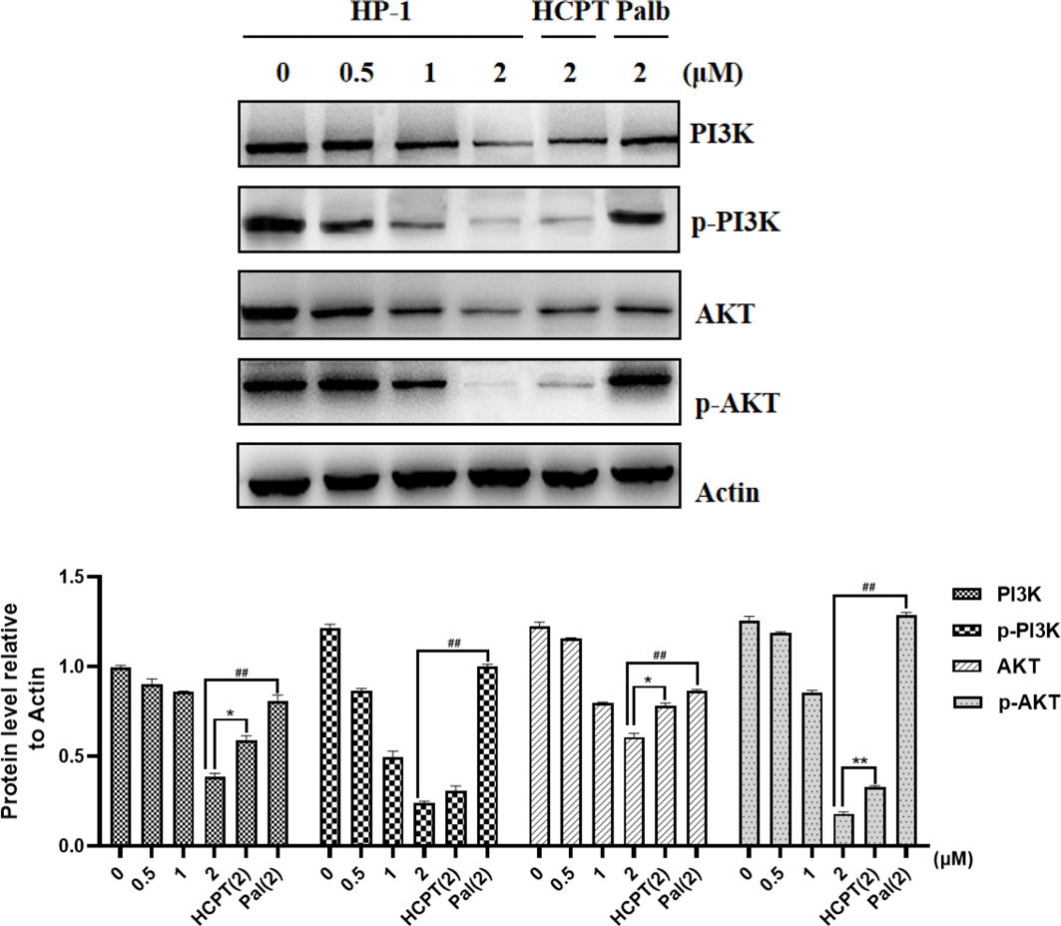
Effect of ***HP-1*** on the expression level of PI3K/AKT pathway-related proteins in NCI-H460 cells. Data shown are the mean ± SEM of three independent experiments (^*^ *p*<0.05, ^**^ *p*<0.01, compared to HCPT; ^##^ *p*<0.01, compared to palbociclib).

As shown in **Figure 8**, after treatment with various concentrations of *HP-1*, 10-HCPT, and palbociclib, we found that the expression of PI3K, AKT, p-PI3K, and p-AKT was decreased in NCI-H460 cells with the increase in compound *HP-1* concentration. These results indicated that *HP-1* could not only inhibit the expression of PI3K and AKT but also inhibit the activation of PI3K and AKT phosphorylation, thus inducing cell apoptosis.

## 4 Conclusion

In this research, three novel compounds *HP-1*, *HP-2*, and *HP-3* were synthesized by molecular docking and chemical preparation. *In vitro* cytotoxicity test results showed that human lung cancer cells were more sensitive to the three drugs, and *HP-1* had the best anti-proliferation effect. The IC_50_ values of *HP-1* on NCI-H460, NCI-H1299, and A549 were 0.96, 2.29, and 3.41 μM, respectively. The inhibitory effects were better than that of palbociclib and 10-HCPT.

Apoptosis assay indicated that the three drugs had significant inhibitory activity on NCI-H460 cells, especially *HP-1*. The effect of *HP-1* was superior to that of 10-HCPT and palbociclib, and its cytotoxicity to normal lung epithelial cells BEAS-2B was much lower than that of 10-HCPT.

Cell cycle analysis elucidated that *HP-1*, *HP-2*, and *HP-3* had obvious cell cycle arrest phenomenon on NCI-H460 cells, especially *HP-1*. The effect of *HP-1* on NCI-H460 cells was better than that of 10-HCPT and palbociclib. Moreover, *HP-1* had little effect on the cell cycle of normal lung epithelial cells BEAS-2B.

Finally, the mechanism of *HP-1*-induced apoptosis and cell cycle arrest of NCI-H460 cells was explored. Western blot analysis showed that *HP-1* induced apoptosis of NCI-H460 cells through PI3K/AKT. Moreover, it reduces the phosphorylation level of Rb protein through the cyclin D-CDK4/6-Rb pathway and finally blocks the cell cycle in the G1 phase.

In conclusion, *HP-1* is a dual target inhibitor of Topo I and CDK 4/CDK6 with good activity. For human lung cancer cells, both the anti-proliferation effect and cell cycle arrest rate of *HP-1* were higher than that of 10-HCPT and palbociclib, and the cytotoxicity of *HP-1* was much lower than that of 10-HCPT. It has broad prospects for development.

## Data availability statement

The original contributions presented in the study are included in the article/**Supplementary Material**. Further inquiries can be directed to the corresponding authors.

## Author contributions

Study design: L-FB and X-YZhu. Study conduct: A-DZ, TL and Y-TZ. Data collection: A-DZ and X-YZ. Data analysis: A-DZ, X-QG, and TL. Data interpretation: A-DZ, H-LY, L-TX, and Y-TZ. Drafting manuscript: TL. Revising manuscript content: TL, X-YZha, and A-DZ. Approving final version of manuscript: TL, D-JW, H-WG, and X-MW. A-DZ takes responsibility for the integrity of the data analysis. All authors contributed to the article and approved the submitted version.

## Funding

This work was supported by the National Natural Science Foundation of China (Grant No. 81970401), the Natural Science Foundation of Jiangsu Province (BK20191122) and Project supported by Medical Science and technology development Foundation, Jiangsu Commission of Health (H2019085), Project supported by Nanjing medical science and technology development fund (YKK21190), the Transportation Science and Technology Project of Jiangsu Province (2021G08).

## Conflict of interest

The authors declare that the research was conducted in the absence of any commercial or financial relationships that could be construed as a potential conflict of interest.

## Publisher’s note

All claims expressed in this article are solely those of the authors and do not necessarily represent those of their affiliated organizations, or those of the publisher, the editors and the reviewers. Any product that may be evaluated in this article, or claim that may be made by its manufacturer, is not guaranteed or endorsed by the publisher.
