# Supplementary material for: Design, synthesis, and anticancer activity of three novel palbociclib derivatives
Source: Front Oncol. 2022 Aug 25;12:959322. doi: 10.3389/fonc.2022.959322 (PMC9453454; doi:10.3389/fonc.2022.959322)
Supplement: Supplementary file 1 [file DataSheet_1.docx]

**Design, synthesis and anticancer activity of three novel palbociclib derivatives**

Tian Li^†,1^, An-Di Zhou^†,1^, Li-Fei Bai^2^, Xiao-Yang Zhang^3^, Yu-Ting Zhou^1^, Hai-Li Yang^1^，Le-Tian Xu^1^，Xin-Qin Guo^1^，Xi-Yu Zhu^1^，Dong-Jin Wang^*,1^，Hong-Wei Gu^*,3^, Xiao-Ming Wang^*,1^

^1^Department of Cardio-Thoracic Surgery, State Key Laboratory of Pharmaceutical Biotechnology, Affiliated Drum Tower Hospital, Medical School of Nanjing University, School of Life Sciences, Nanjing University, Nanjing 210023, China

^2^Jiangsu Key Laboratory of Biofunction Molecule, School of Life Science, and Chemical Engineering, Jiangsu Second Normal University, Nanjing 210013, China.

^3^Central Laboratory, Nanjing Integrated Traditional Chinese and Western Medicine Hospital Affiliated with Nanjing University of Chinese Medicine, Nanjing, 210014, China.

^*^Address correspondence to: Xiao-Ming Wang, State Key Laboratory of Pharmaceutical Biotechnology, School of Life Sciences, Nanjing University, Nanjing 210023, PR China. E-mail: [wangxm07@nju.edu.cn](mailto:wangxm07@nju.edu.cn)

^*^Address correspondence to: Dong-Jin Wang, Department of Cardio-Thoracic Surgery, State Key Laboratory of Pharmaceutical Biotechnology, Affiliated Drum Tower Hospital, Medical School of Nanjing University, Nanjing University, Nanjing 210023, PR China. E-mail: wangdongjin@njglyy.com

^*^Address correspondence to: Hong-Wei Gu, Central Laboratory, Nanjing Integrated Traditional Chinese and Western Medicine Hospital Affiliated with Nanjing University of Chinese Medicine, Nanjing, 210014, China. E-mail: [hongweiigu@hotmail.com](mailto:hongweiigu@hotmail.com)

^†^These authors have contributed equally to this work and share first authorship.


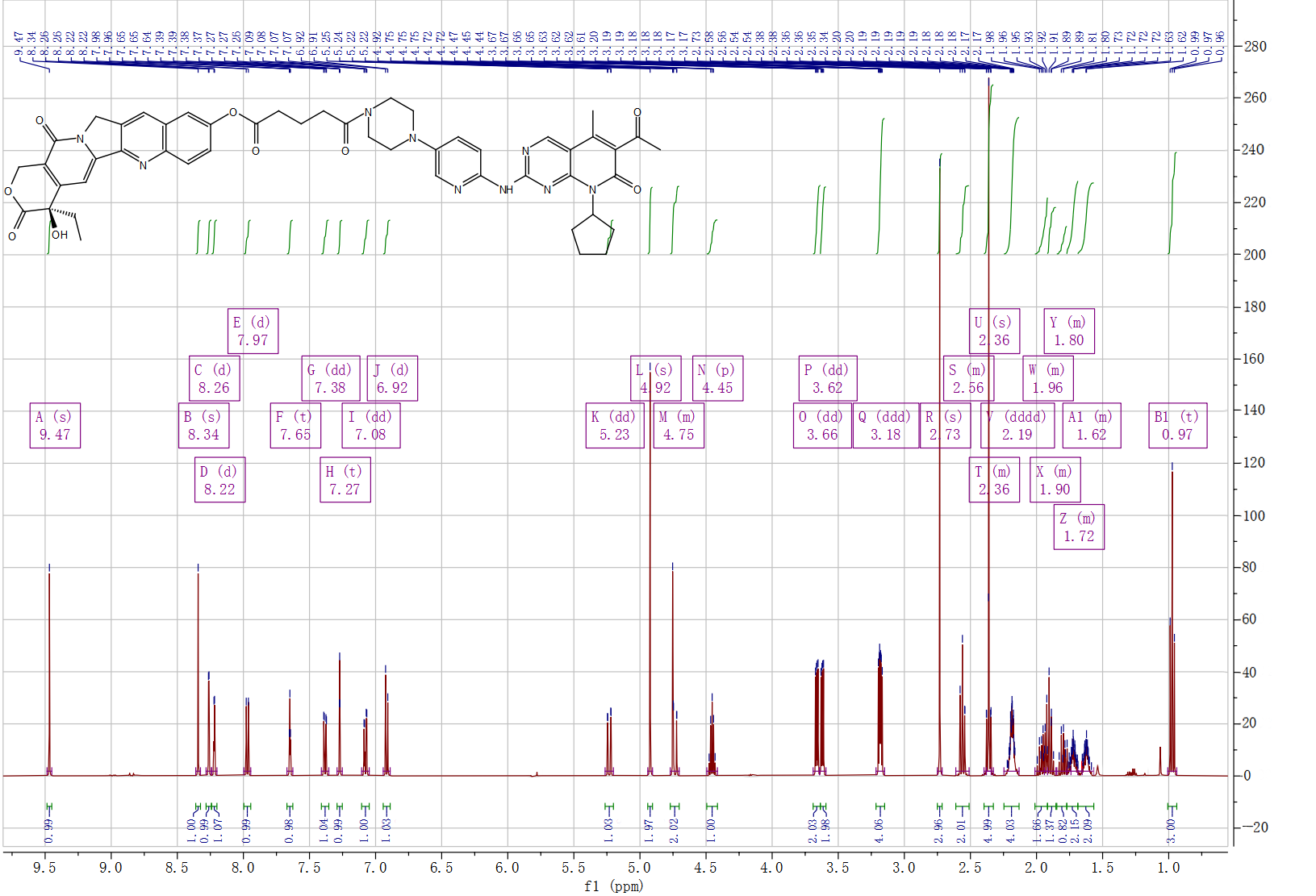


**Figure S1** The ^1^H NMR spectrum of the compound **HP-1**

**
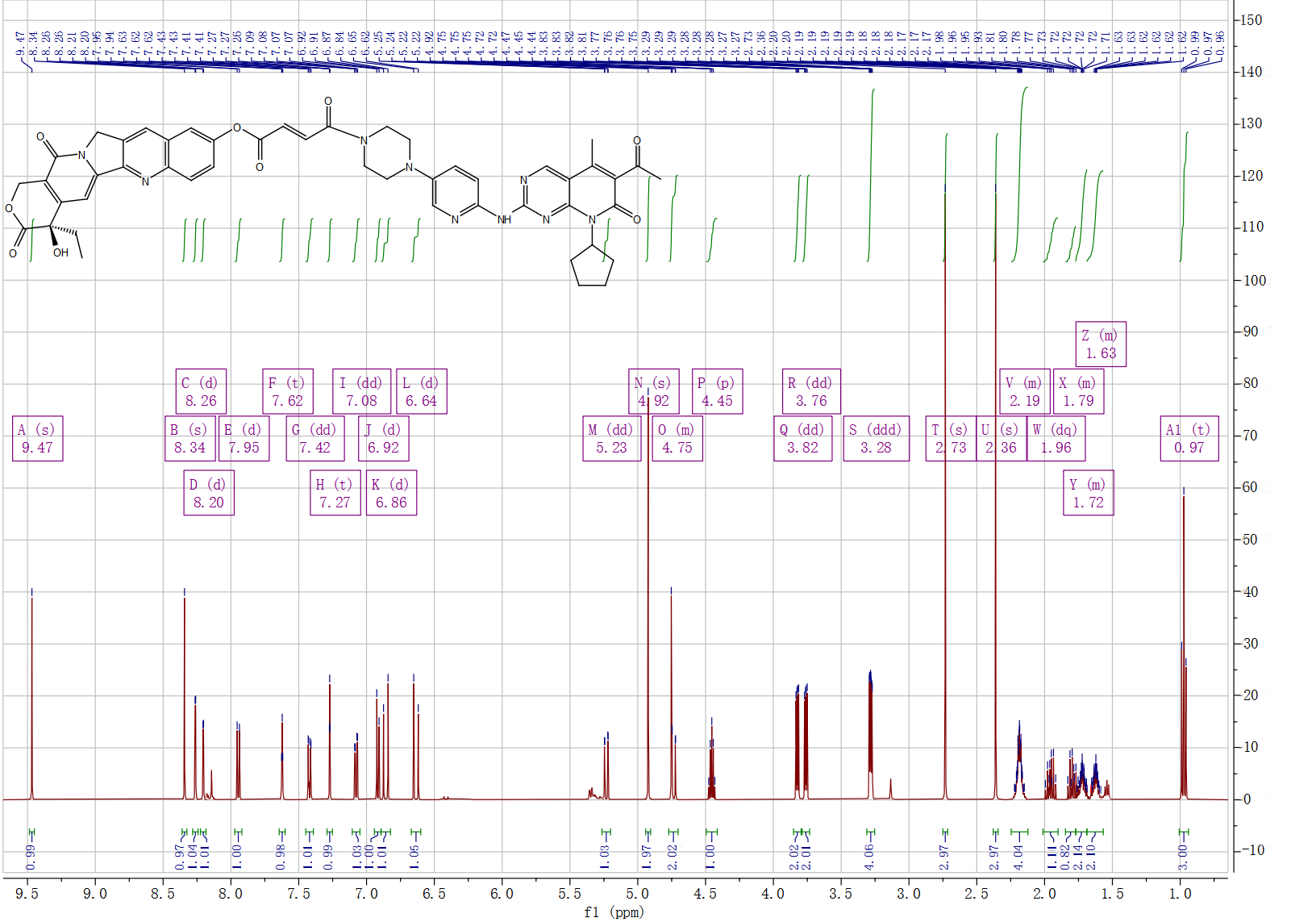
**

**Figure S2** The ^1^H NMR spectrum of the compound **HP-2**


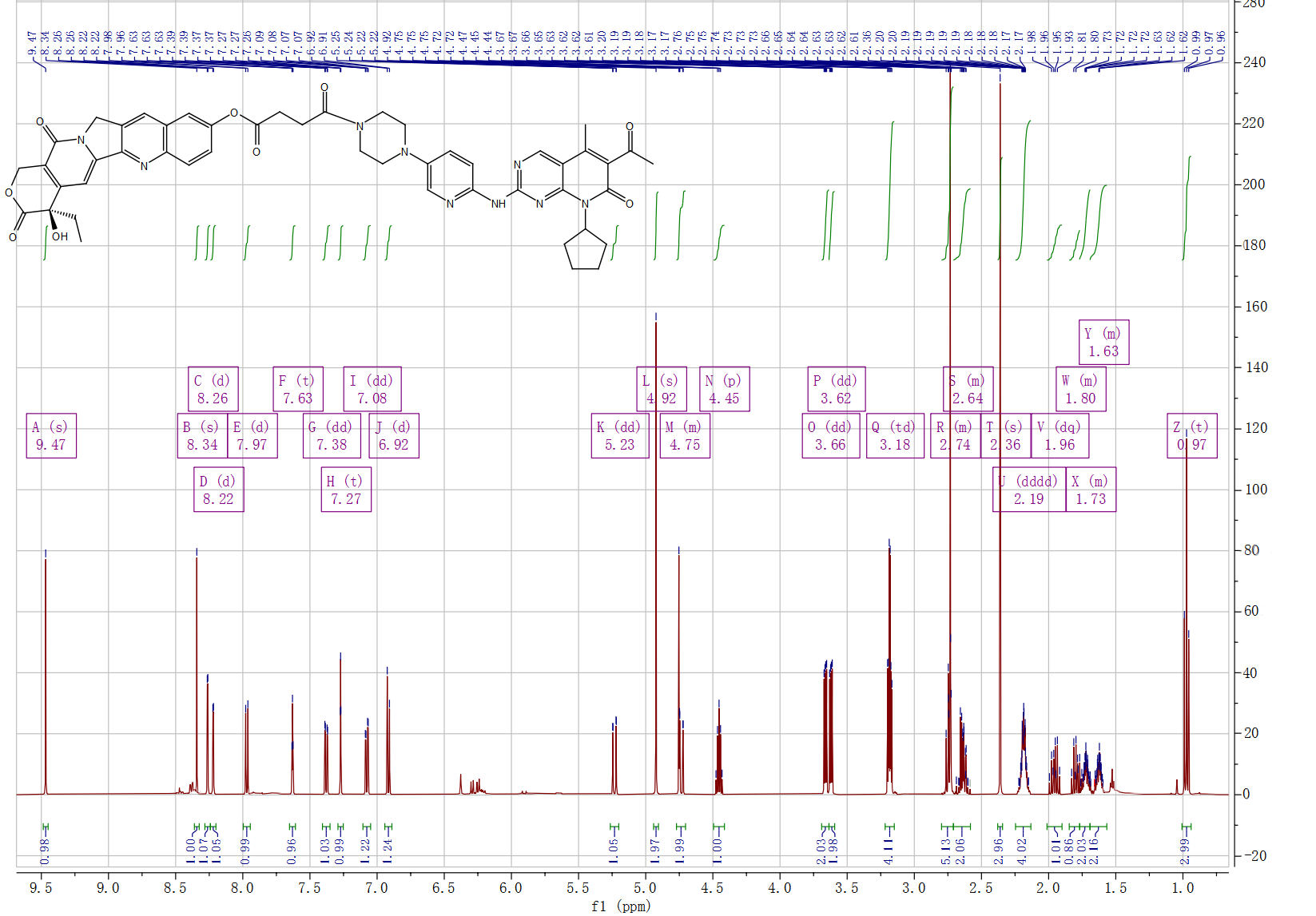


**Figure S3** The ^1^H NMR spectrum of the compound **HP-3**

**
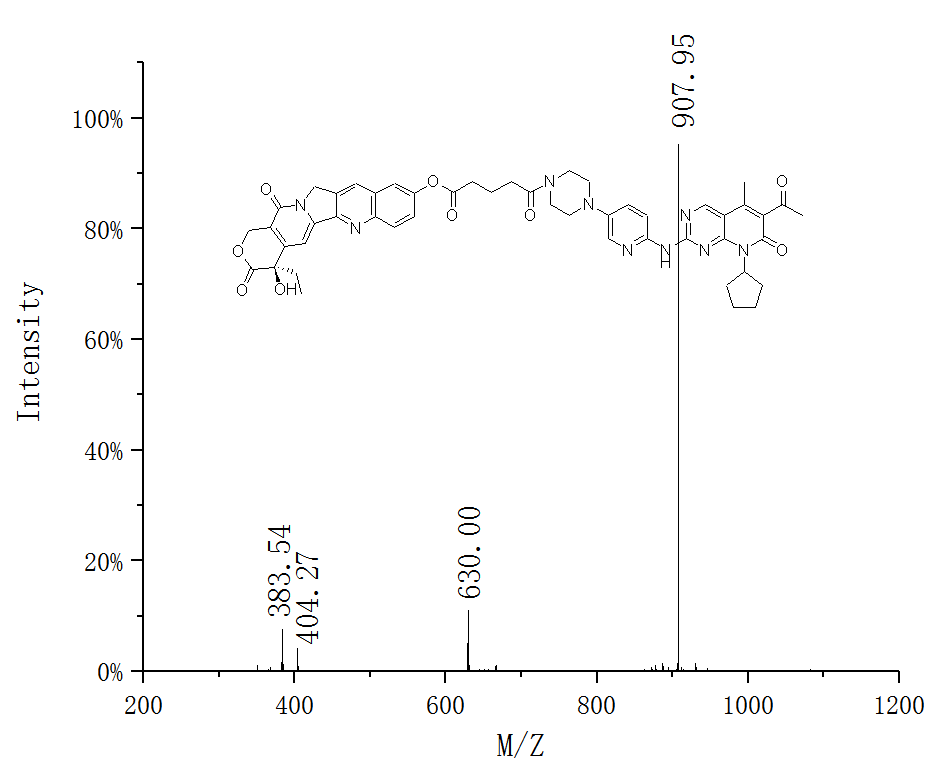
**

**Figure S4** The mass spectrometry spectrum of the compound **HP-1**


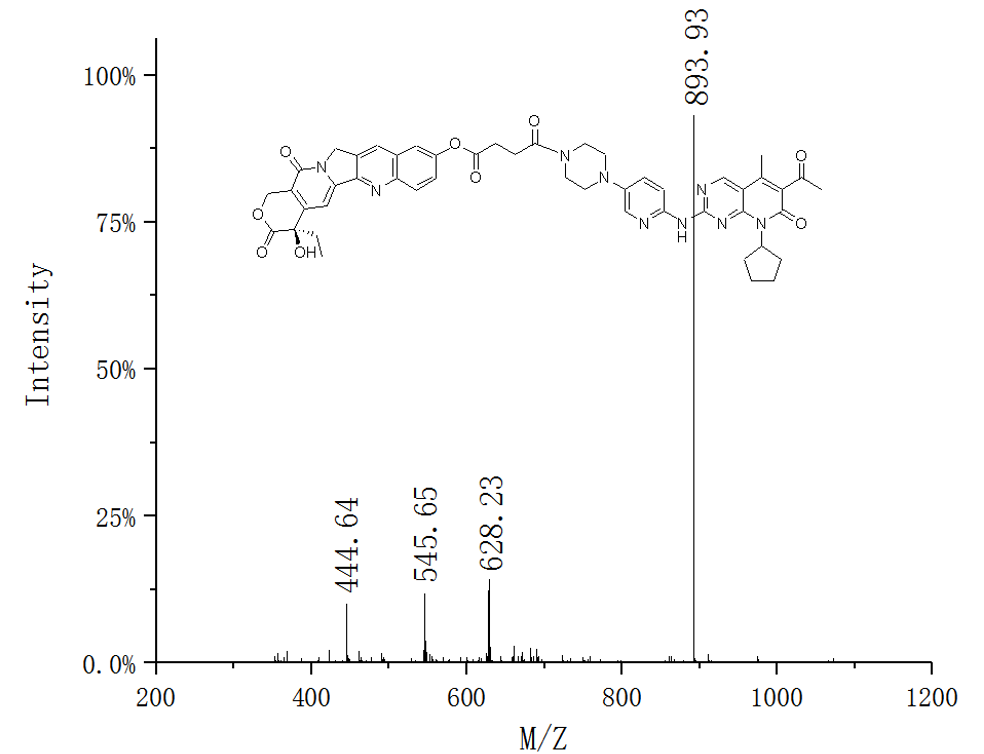


**Figure S5** The mass spectrometry of the compound **HP-2**


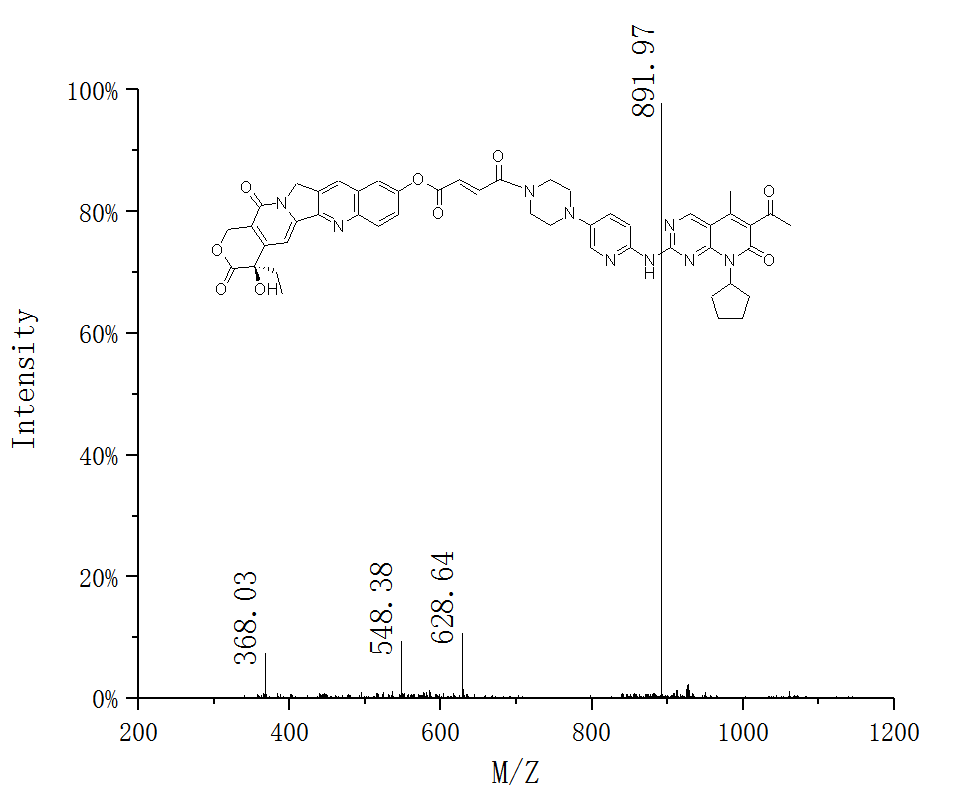


**Figure S6** The mass spectrometry spectrum of the compound **HP-3**


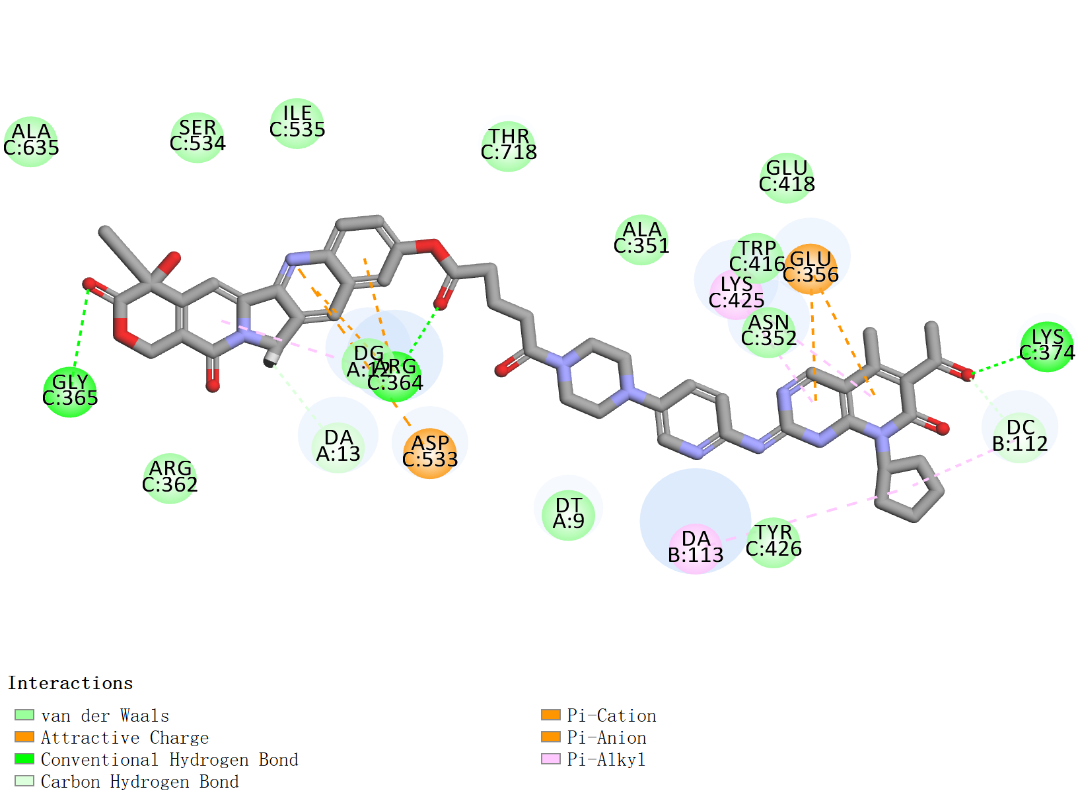


**(B)**

**(A)**


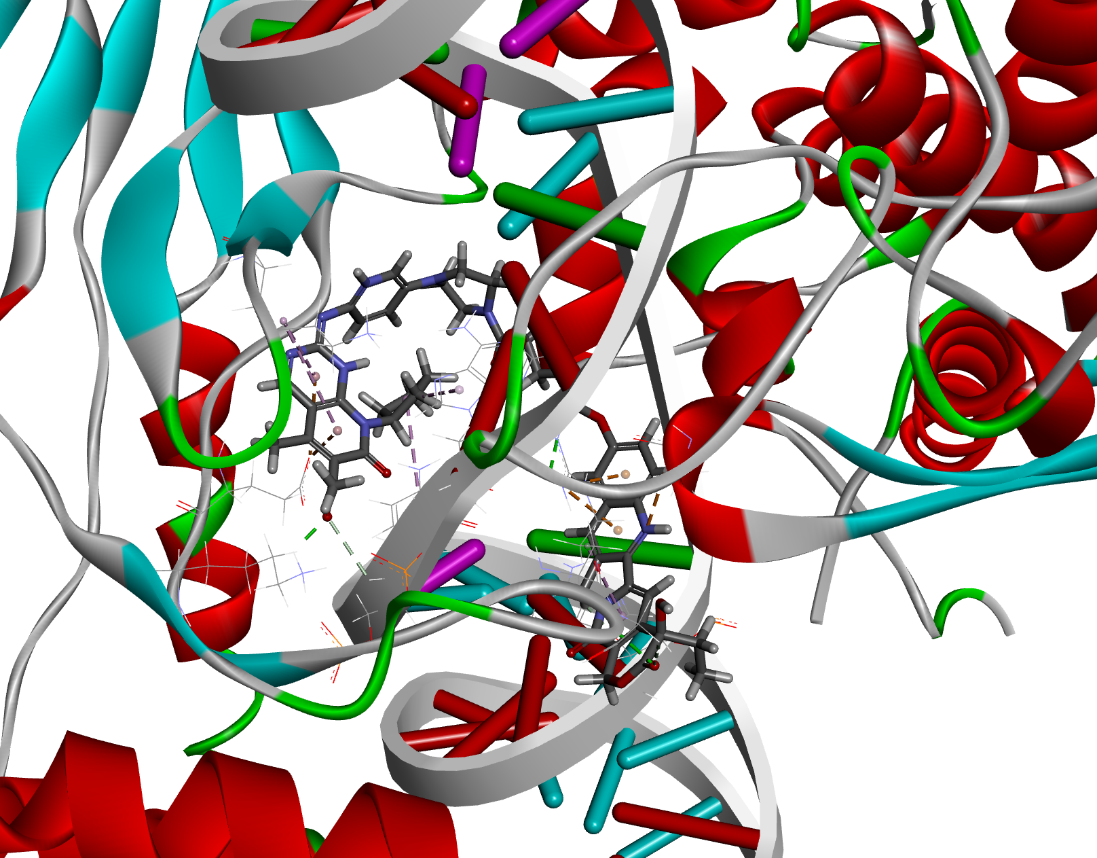


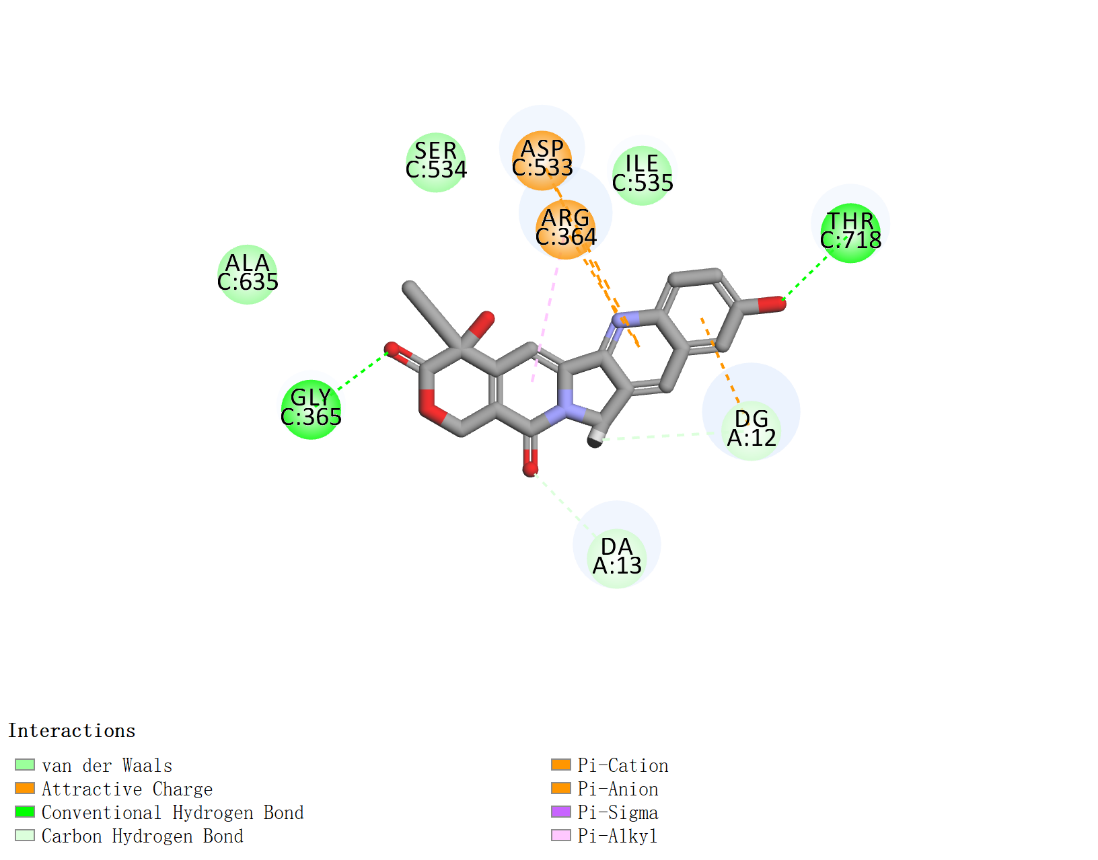


**(D)**

**(C)**


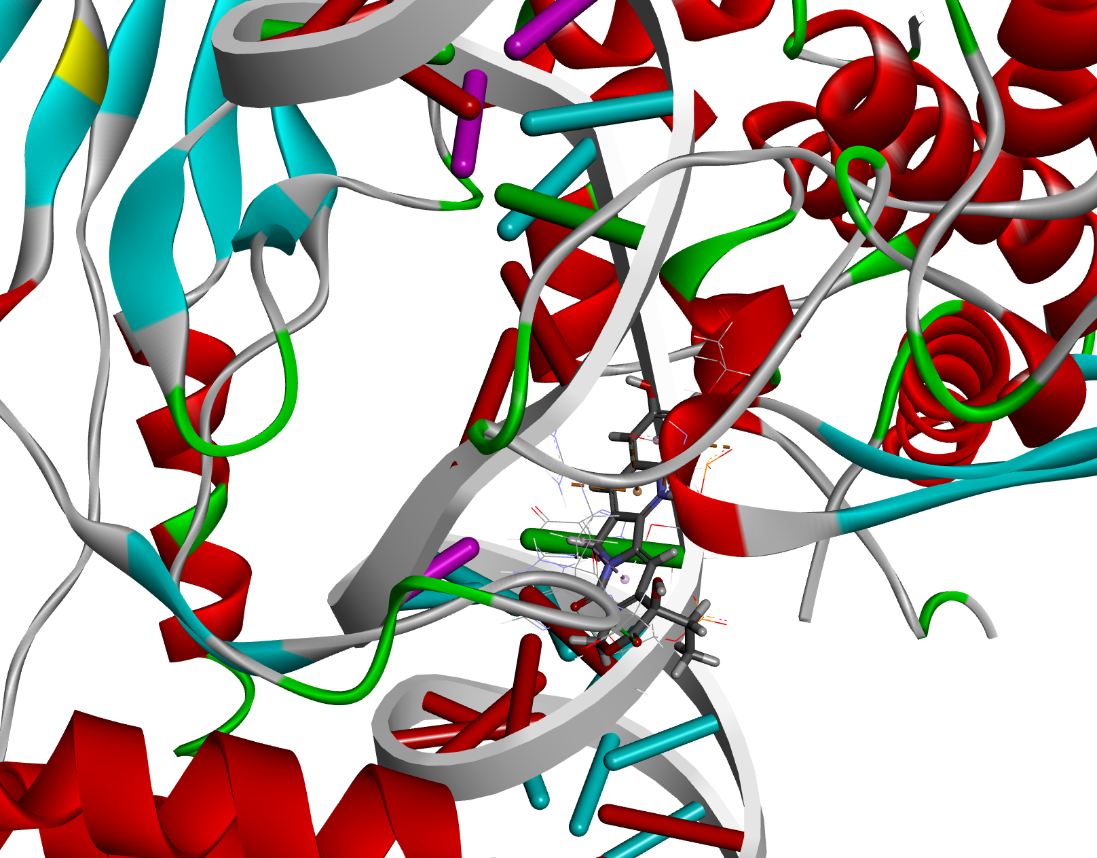


**Figure S7.** Molecular docking of the compound HP-1 and 10-hydroxycamptothecin with the active site of Topo I (PDB: 1RR8). **(A)** Two dimensional diagram of interactions of **HP-1** with the amino acid residues at Topo I. **(B)** Three dimensional diagram of interactions of **HP-1** with Topo I. **(C)** Two dimensional diagram of interactions of HCPT with the amino acid residues at Topo I. **(D)** Three dimensional diagram of interactions of HCPT with Topo I.


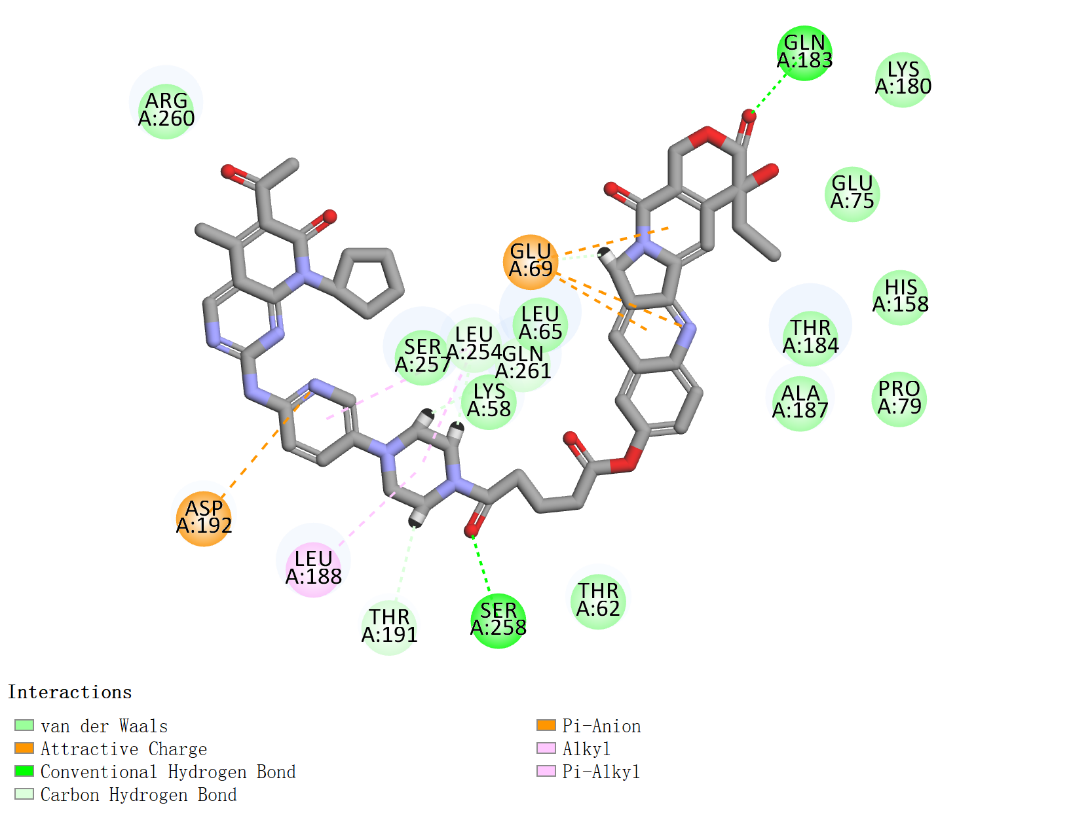


**(A)**


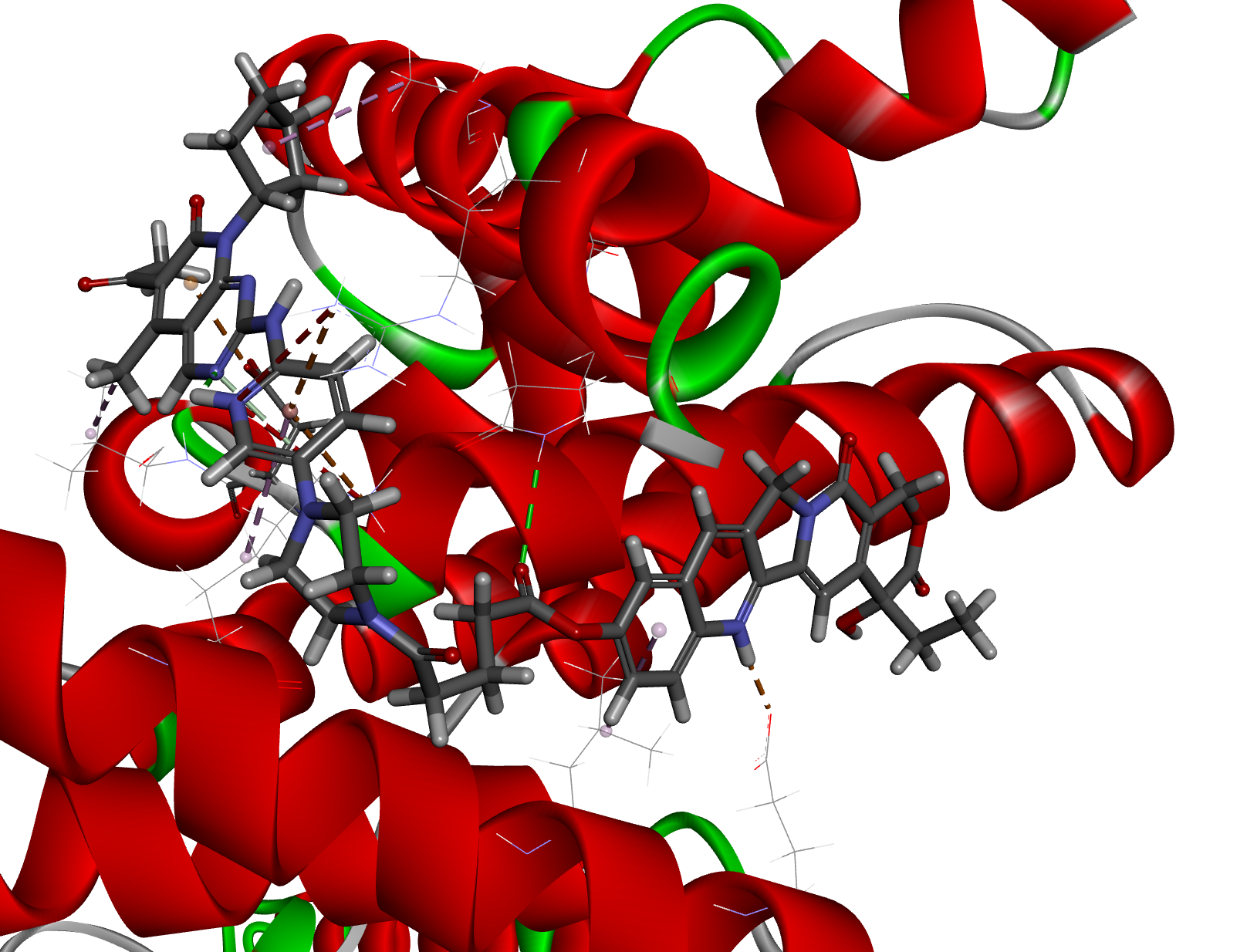


**(B)**


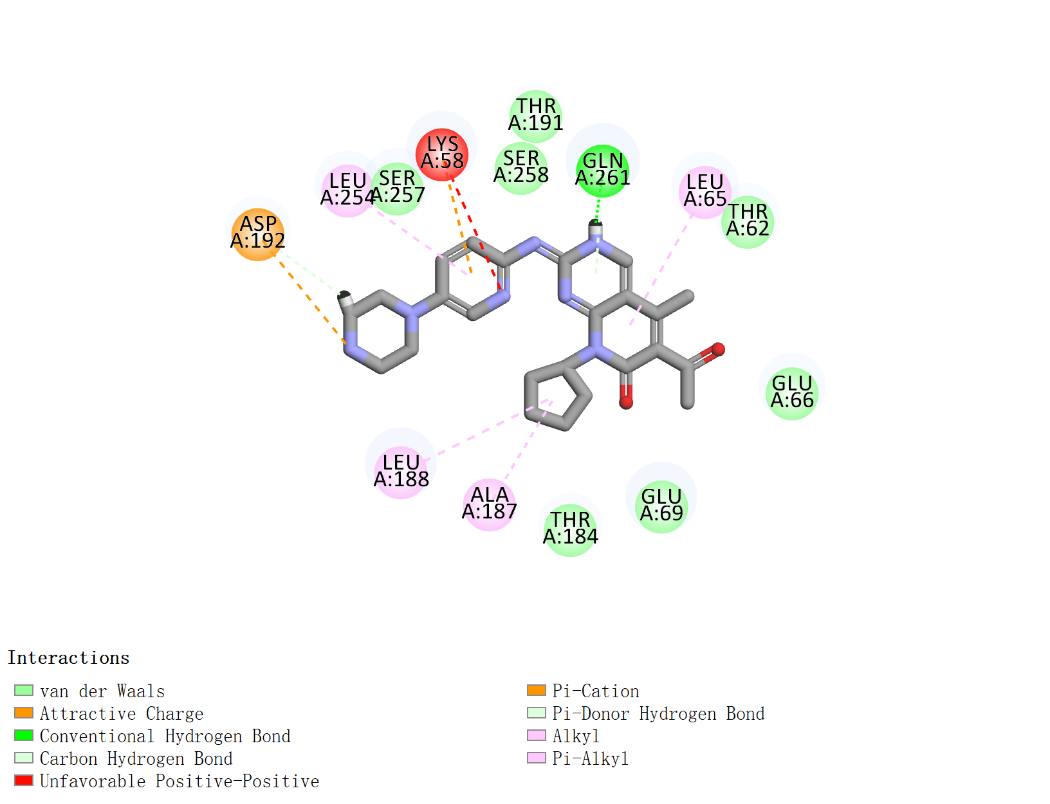


**(C)**


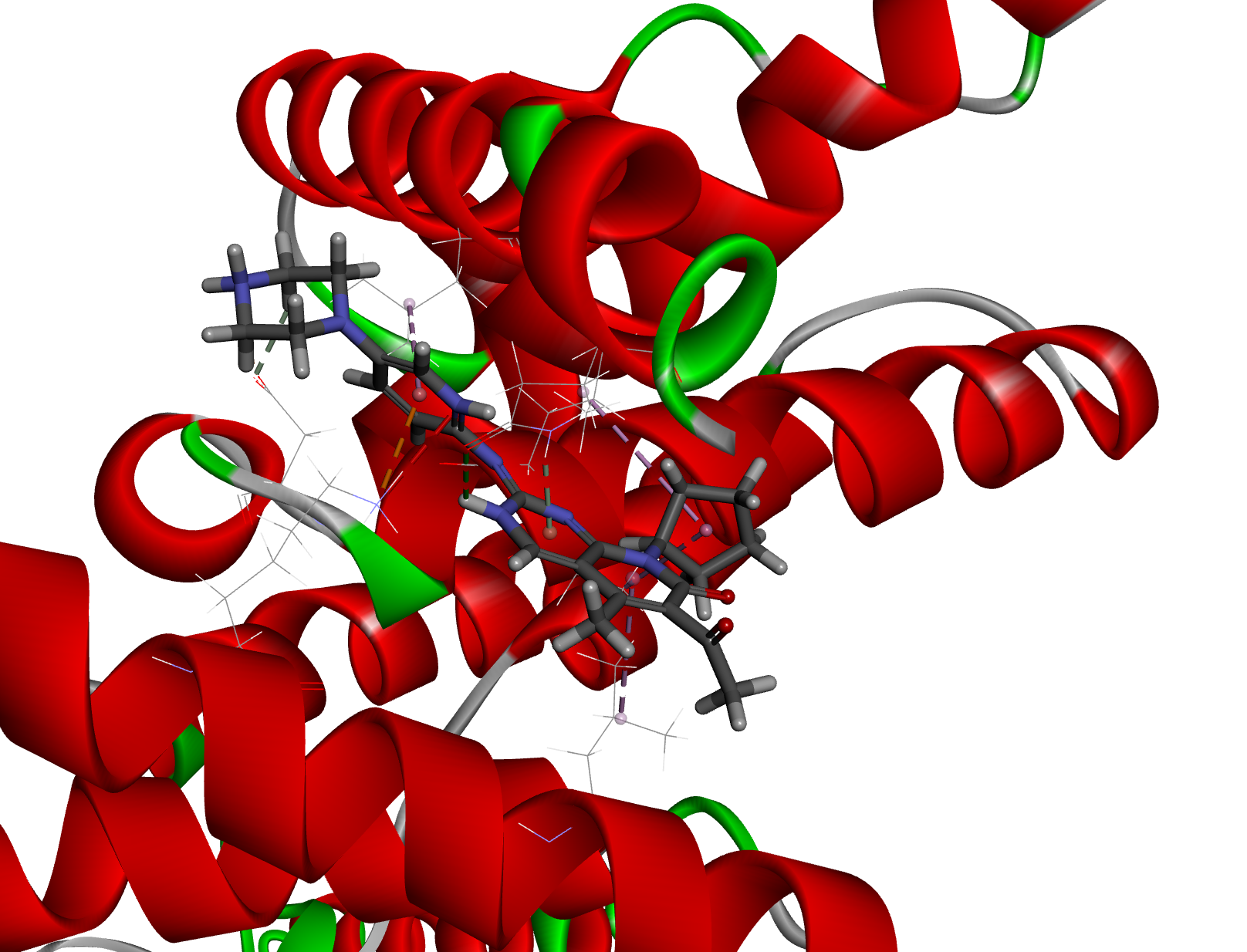


**(D)**

**Figure S8.** Molecular docking of the compound HP-1 and palbociclib with the active site of CDK4 (PDB: 2W99). **(A)** Two dimensional diagram of interactions of **HP-1** with the amino acid residues at CDK4. **(B)** Three dimensional diagram of interactions of **HP-1** with CDK4. **(C)** Two dimensional diagram of interactions of palbociclib with the amino acid residues at CDK4. **(D)** Three dimensional diagram of interactions of palbociclib with CDK4.


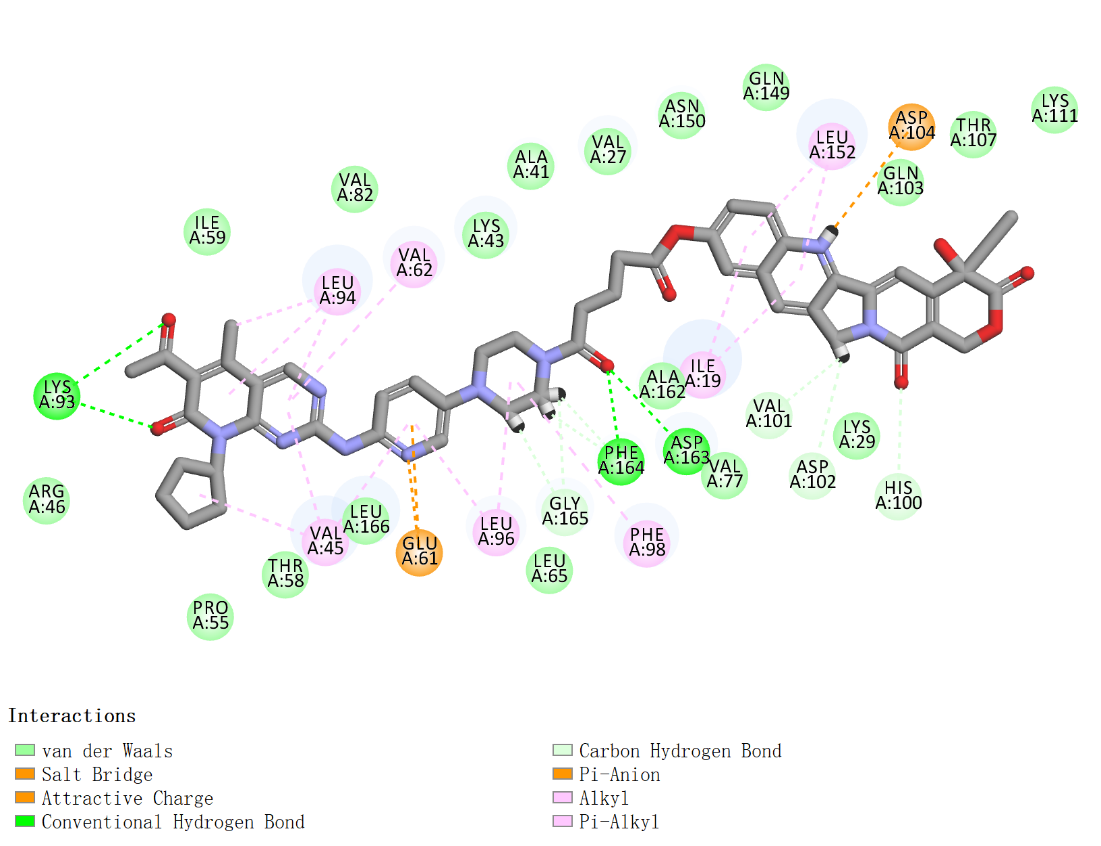


**(A)**


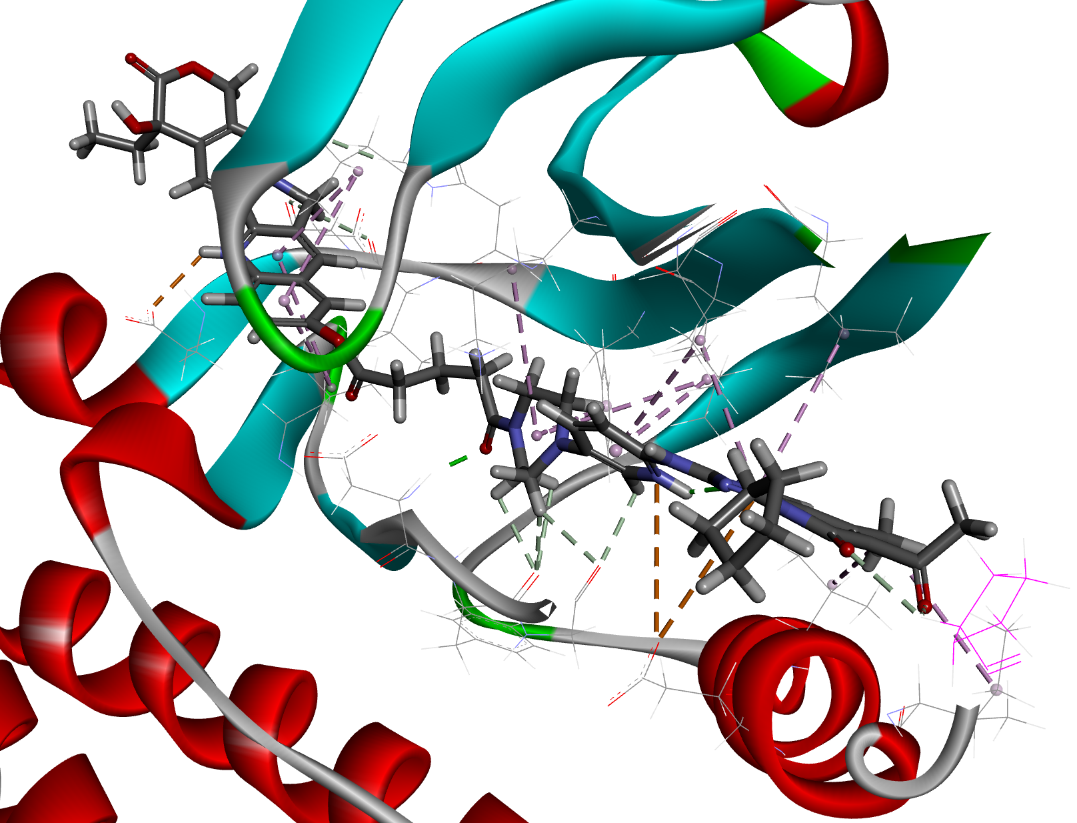


**(B)**


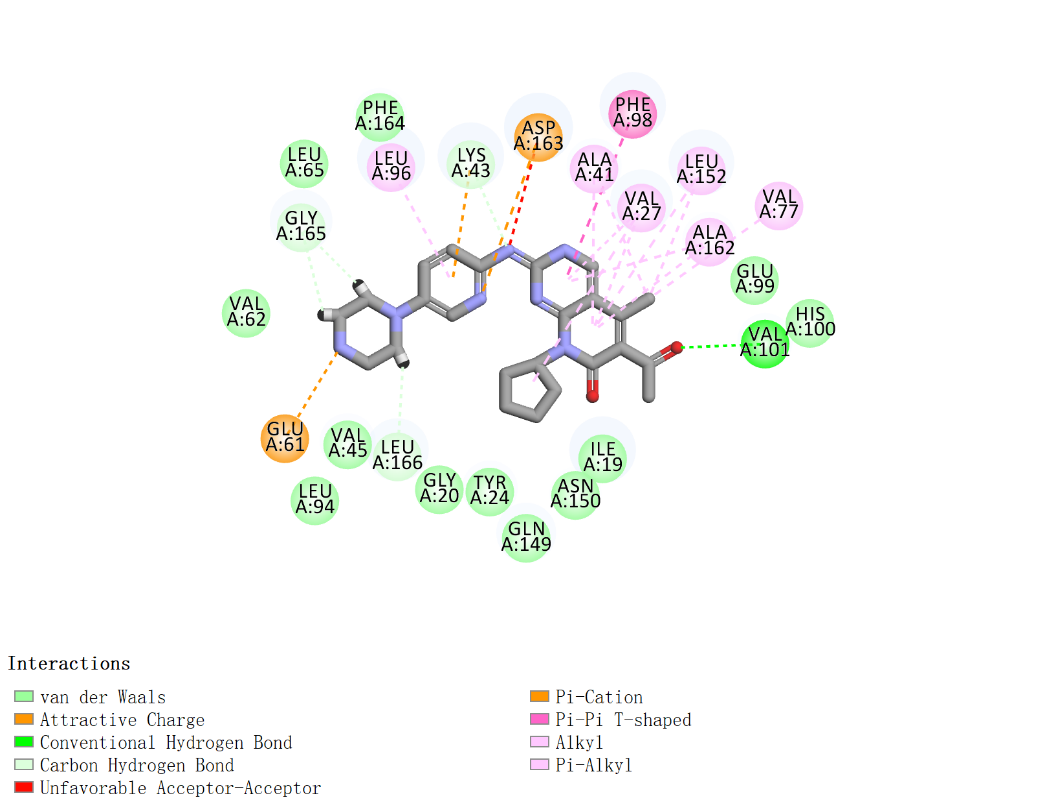


**(C)**


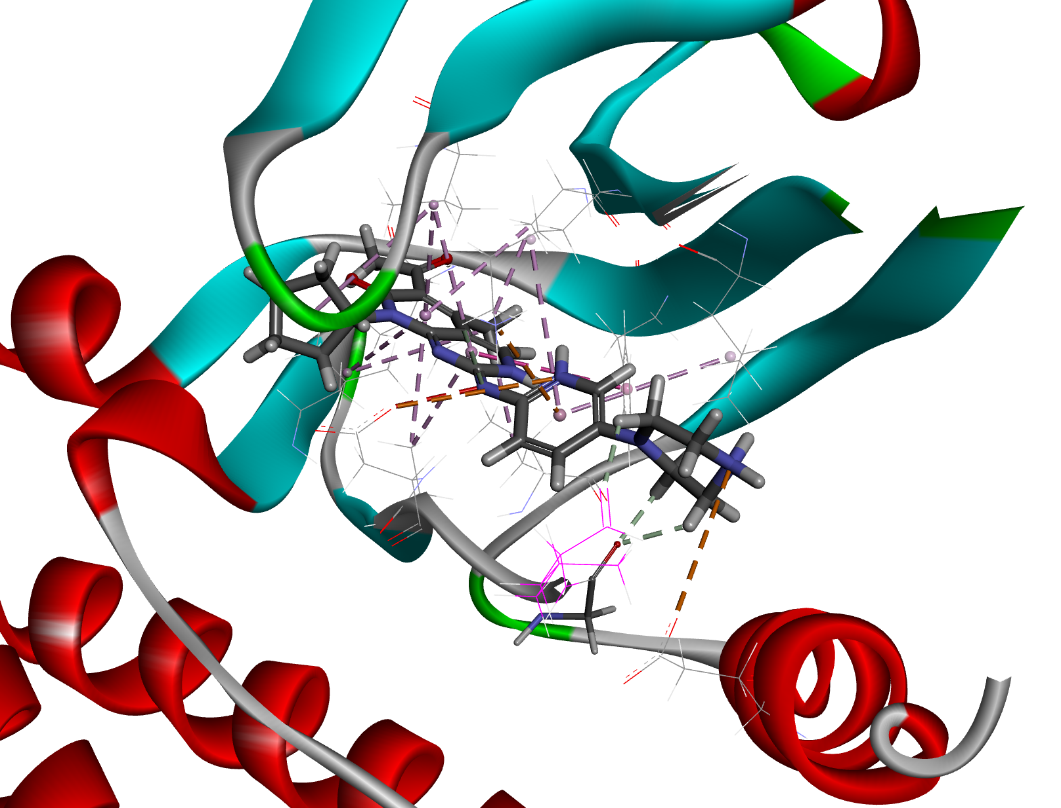


**(D)**

**Figure S9.** Molecular docking of the compound HP-1 and palbociclib with the active site of CDK6 (PDB: 5L2I). **(A)** Two dimensional diagram of interactions of **HP-1** with the amino acid residues at CDK6. **(B)** Three dimensional diagram of interactions of **HP-1** with CDK6. **(C)** Two dimensional diagram of interactions of palbociclib with the amino acid residues at CDK6. **(D)** Three dimensional diagram of interactions of palbociclib with CDK6.

**
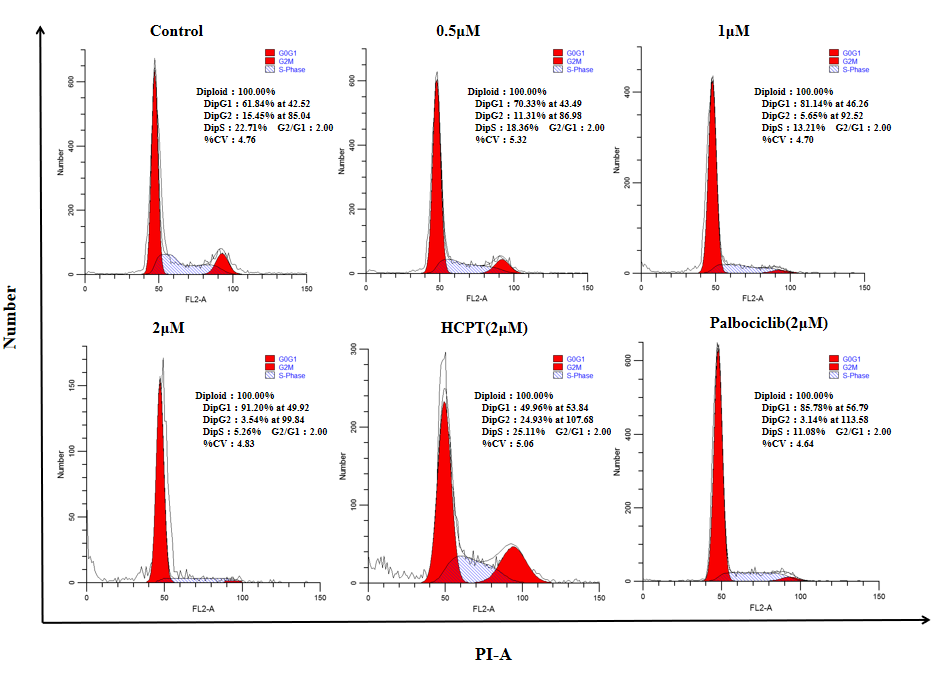
**


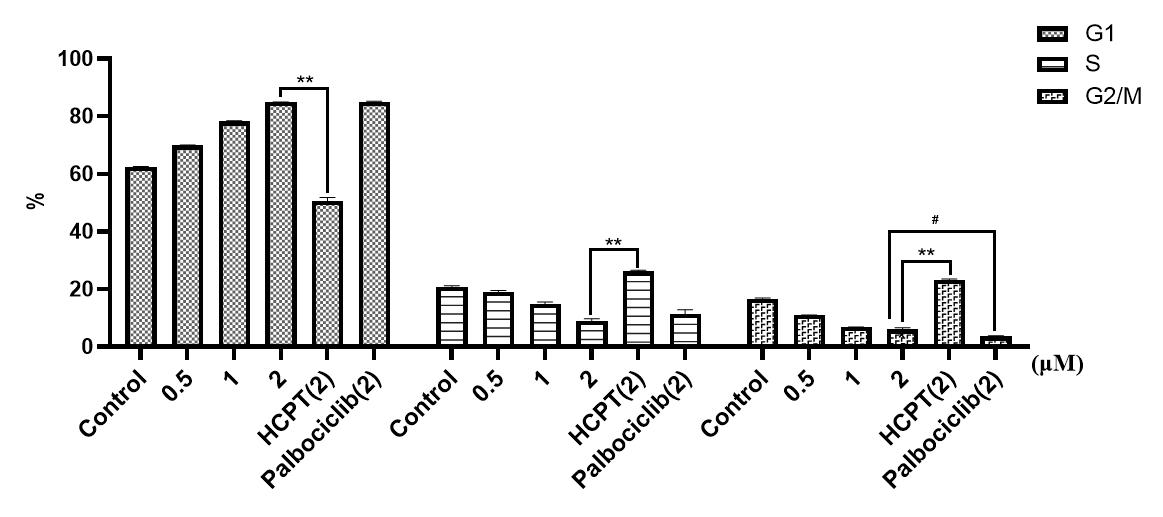


**Figure S10.** Effect of **HP-2** on the cell cycle of NCI-H460 cells in concentration dependence. Data shown are the mean ± SD. of three independent experiments (^*^*p* <0.05, ^**^ *p* <0.01, compared to HCPT; ^#^ *p* <0.05，^##^ *p* <0.01, compared to palbociclib).


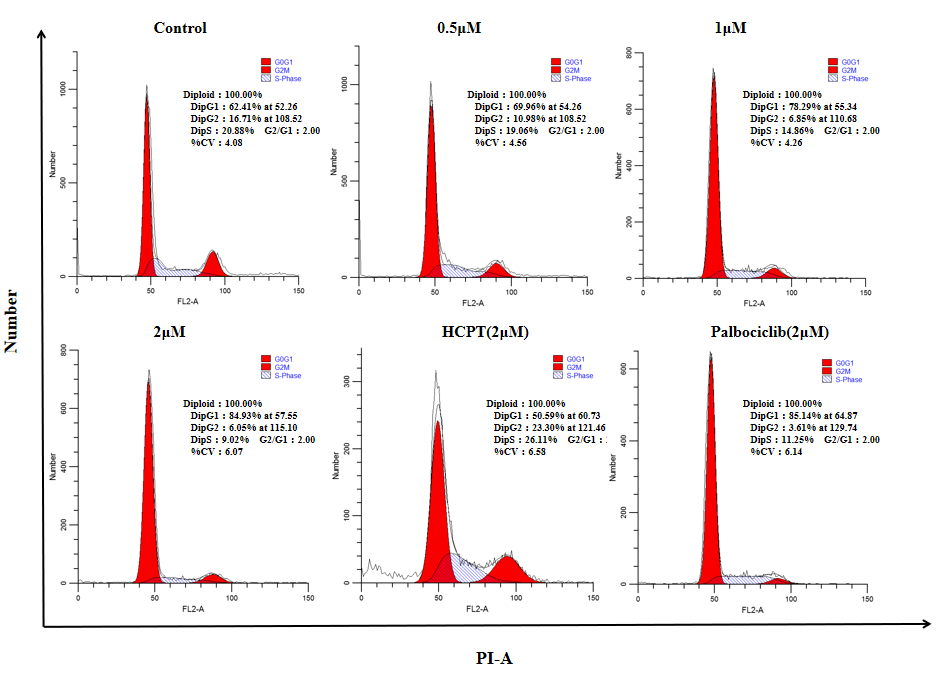


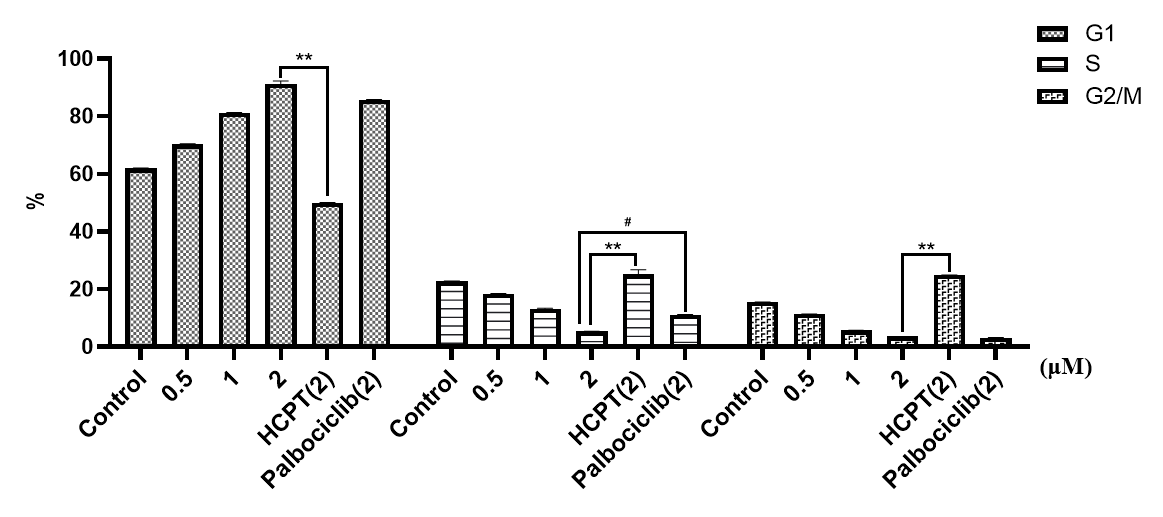


**Figure S11** Effect of **HP-3** on the cell cycle of NCI-H460 cells in concentration dependence. Data shown are the mean ± SD. of three independent experiments (^*^*p* <0.05, ^**^ *p* <0.01, compared to HCPT; ^#^ *p* <0.05，^##^ *p* <0.01, compared to palbociclib).


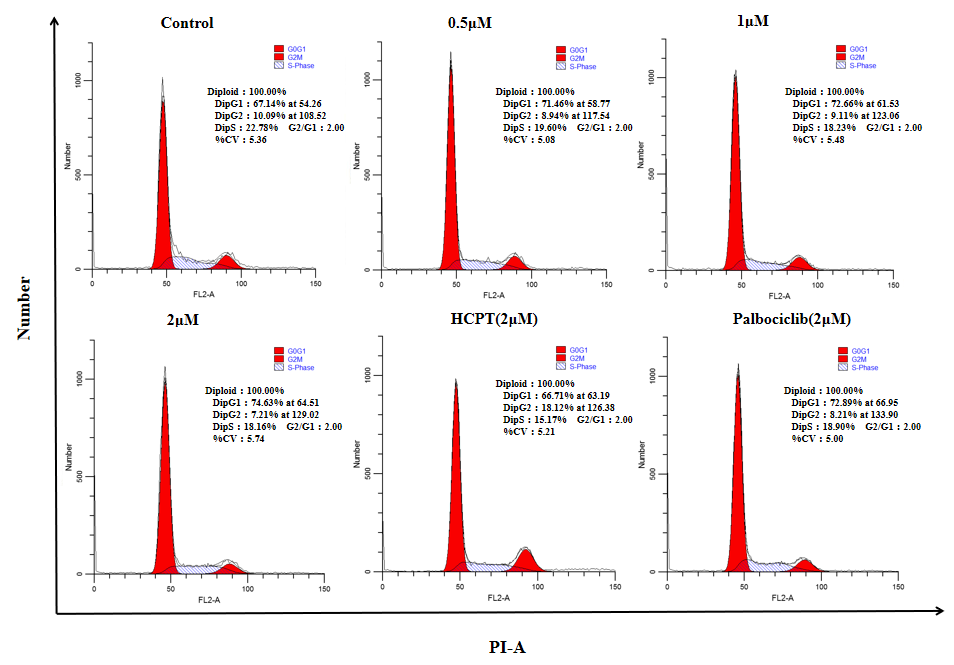


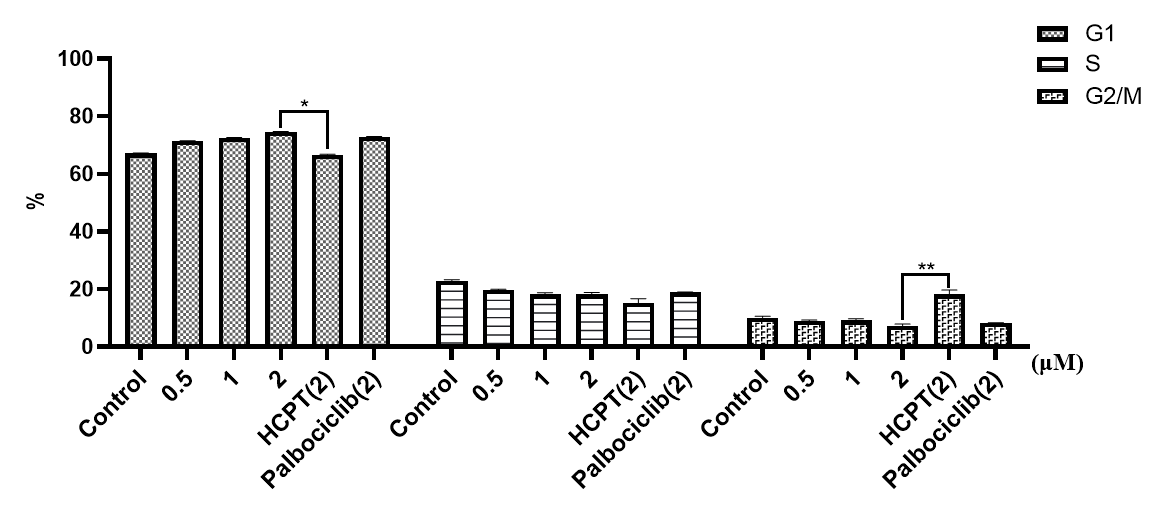


**Figure S12.** Effect of **HP-1** on the cell cycle of Beas-2B cells in concentration dependence. Data shown are the mean ± SD. of three independent experiments (^*^*p* <0.05, ^**^ *p* <0.01, compared to HCPT; ^#^ *p* <0.05，^##^ *p* <0.01, compared to palbociclib).


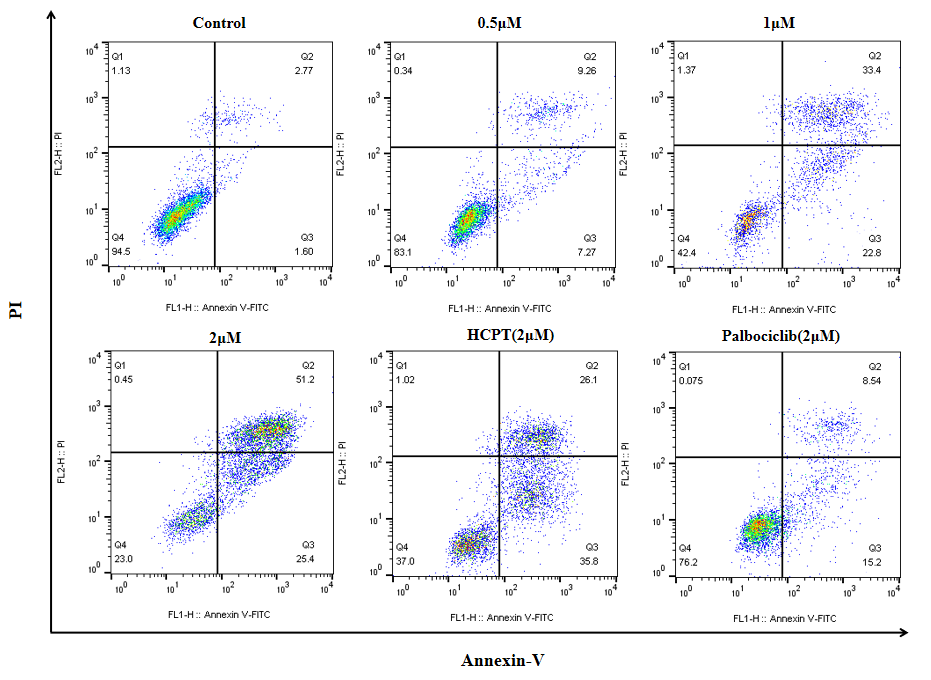


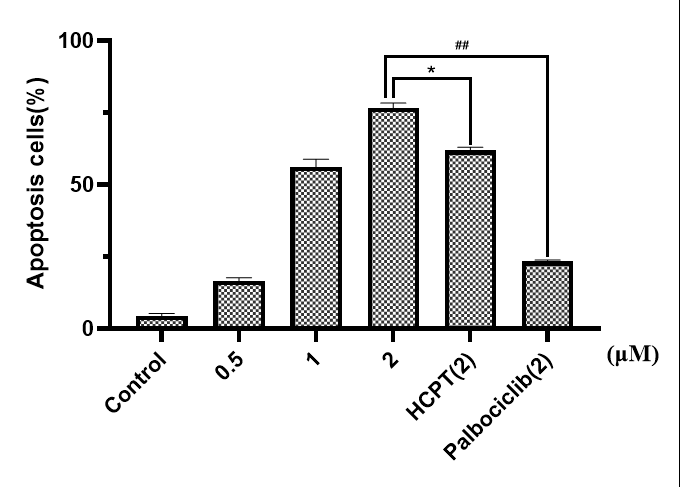


**Figure S13.** Effect of **HP-2** on the cell apoptosis of NCI-H460 cells in concentration dependence. Data shown are the mean ± SD. of three independent experiments (^*^*p*<0.05, ^**^ *p* <0.01, compared to HCPT; ^#^ *p* <0.05，^##^ *p* <0.01, compared to palbociclib).


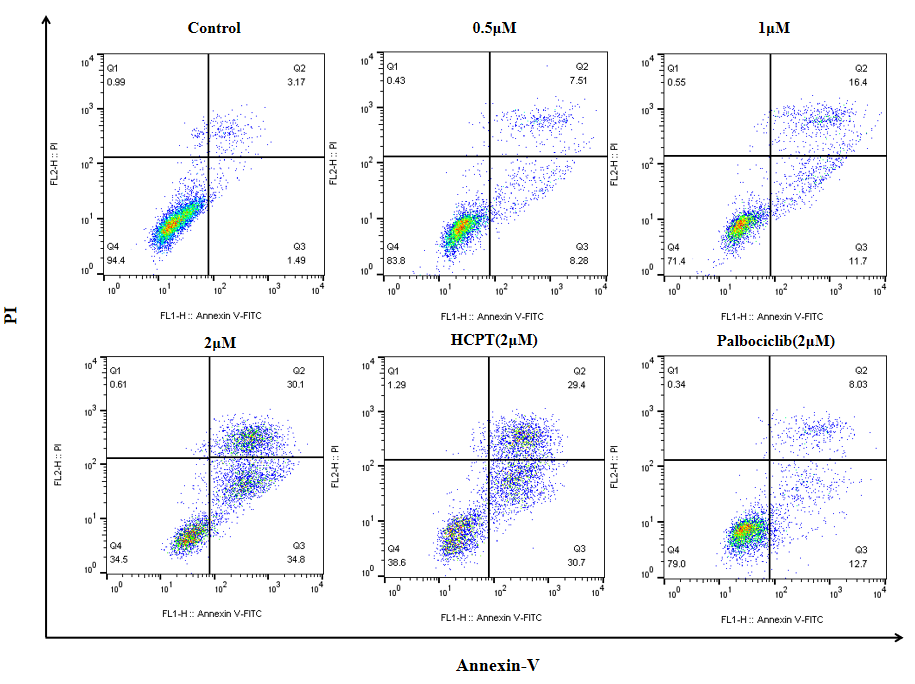


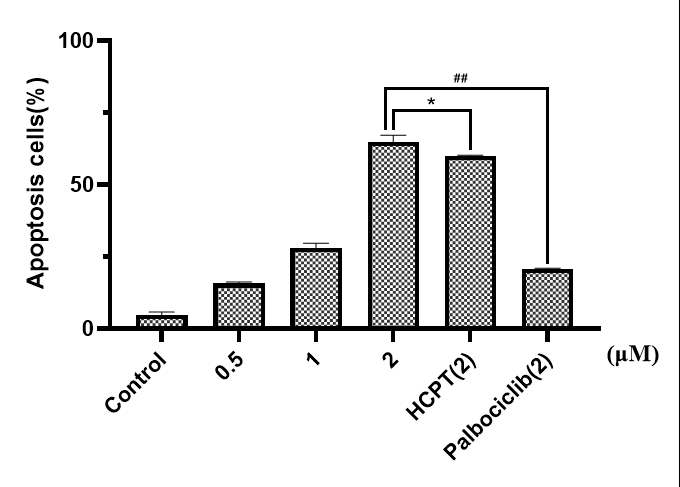


**Figure S14.** Effect of **HP-3** on the cell apoptosis of NCI-H460 cells in concentration dependence. Data shown are the mean ± SD. of three independent experiments (^*^*p* <0.05, ^**^ *p* <0.01, compared to HCPT; ^#^ *p* <0.05，^##^ *p* <0.01, compared to palbociclib).


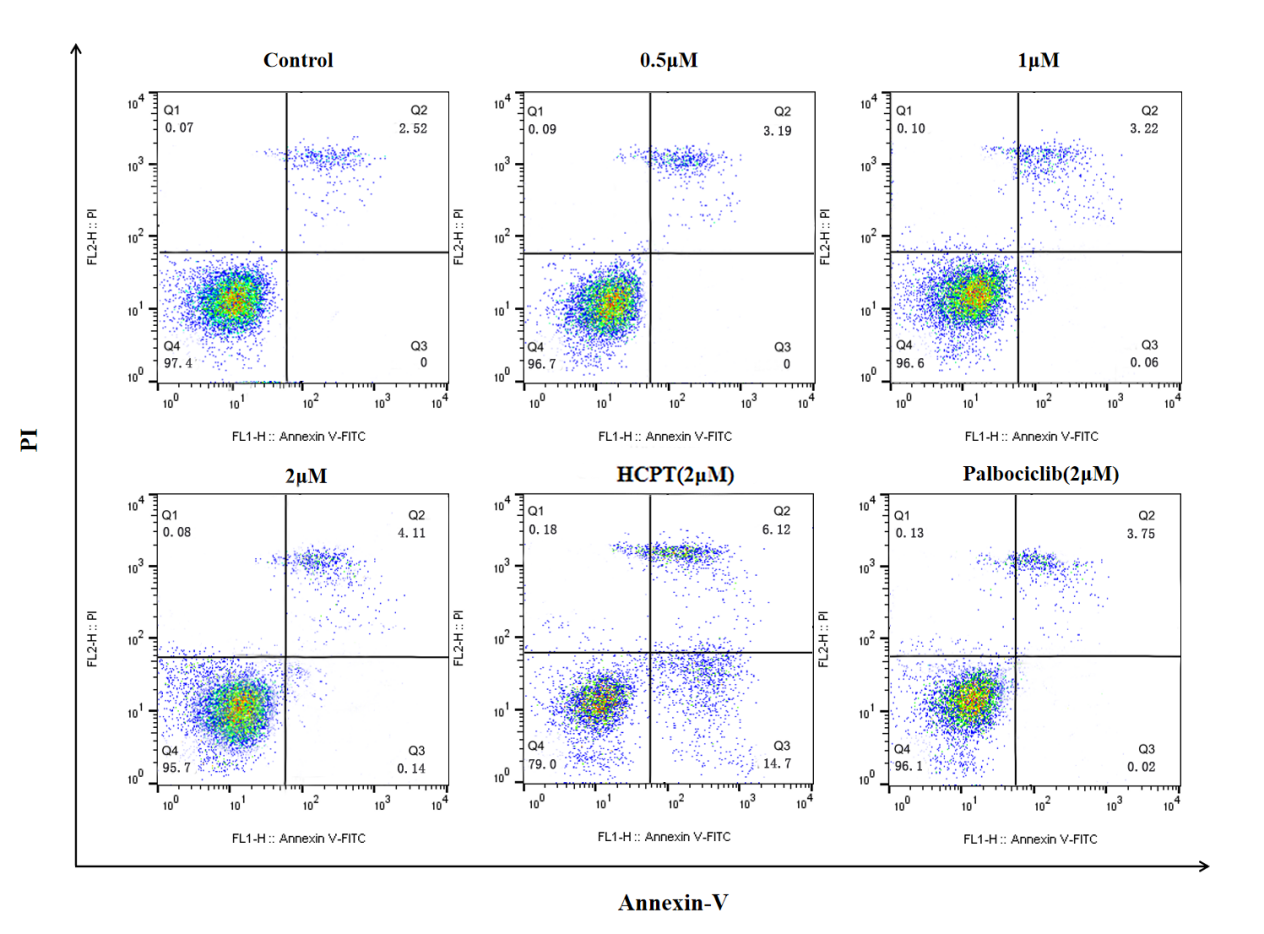


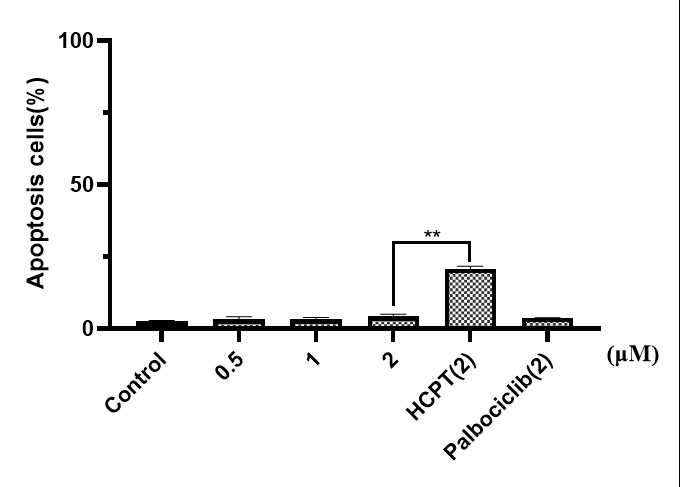


**Figure S15.** Effect of **HP-1** on the apoptosis of Beas-2B cells in concentration dependence. Data shown are the mean ± SD. of three independent experiments (^*^*p* <0.05, ^**^ *p* <0.01, compared to HCPT; ^#^ *p* <0.05，^##^ *p* <0.01, compared to palbociclib).
